# Compositionally Graded Absorber for Efficient and Stable Near‐Infrared‐Transparent Perovskite Solar Cells

**DOI:** 10.1002/advs.201700675

**Published:** 2018-01-05

**Authors:** Fan Fu, Stefano Pisoni, Thomas P. Weiss, Thomas Feurer, Aneliia Wäckerlin, Peter Fuchs, Shiro Nishiwaki, Lukas Zortea, Ayodhya N. Tiwari, Stephan Buecheler

**Affiliations:** ^1^ Laboratory for Thin Films and Photovoltaics Empa‐Swiss Federal Laboratories for Materials Science and Technology Ueberlandstrasse 129 CH‐8600 Duebendorf Switzerland

**Keywords:** compositional grading, NIR‐transparent perovskite solar cells, operational stability, partial ion‐exchange, tandem solar cells

## Abstract

Compositional grading has been widely exploited in highly efficient Cu(In,Ga)Se_2_, CdTe, GaAs, quantum dot solar cells, and this strategy has the potential to improve the performance of emerging perovskite solar cells. However, realizing and maintaining compositionally graded perovskite absorber from solution processing is challenging. Moreover, the operational stability of graded perovskite solar cells under long‐term heat/light soaking has not been demonstrated. In this study, a facile partial ion‐exchange approach is reported to achieve compositionally graded perovskite absorber layers. Incorporating compositional grading improves charge collection and suppresses interface recombination, enabling to fabricate near‐infrared‐transparent perovskite solar cells with power conversion efficiency of 16.8% in substrate configuration, and demonstrate 22.7% tandem efficiency with 3.3% absolute gain when mechanically stacked on a Cu(In,Ga)Se_2_ bottom cell. Non‐encapsulated graded perovskite device retains over 93% of its initial efficiency after 1000 h operation at maximum power point at 60 °C under equivalent 1 sun illumination. The results open an avenue in exploring partial ion‐exchange to design graded perovskite solar cells with improved efficiency and stability.

## 1 Introduction

Thin film perovskite (ABX_3_, A = Cs^+^, [CH_3_NH_3_]^+^ (MA^+^), [CH(NH_2_)_2_]^+^ (FA^+^); B = Pb, Sn; X = Cl, Br, I) solar cells have gained considerable attention due to the high performance, easy processing and potentially low‐cost manufacturing.^1^ Benefiting from outstanding optoelectronic properties, such as high absorption coefficient,^2^ large carrier mobility and long carrier diffusion length,^3^, ^4^ and unique defect properties,^5^ the power conversion efficiency of perovskite solar cells has rapidly increased from 3.8% to over 22% by engineering charge transporting layers,^6^, ^7^, ^8^, ^9^ optimizing the absorber composition and deposition techniques.^10^, ^11^, ^12^, ^13^, ^14^, ^15^, ^16^, ^17^, ^18^, ^19^ The wide bandgap with flexibility to tune over a broad energy range renders perovskite solar cells ideal candidates for top cells in tandem applications with narrow bandgap bottom cells,^20^, ^21^ such as crystalline‐Si,^22^, ^23^, ^24^, ^25^ thin film Cu(In,Ga)Se_2_ (CIGS),^26^, ^27^, ^28^, ^29^, ^30^, ^31^ Sn‐based halide perovskites,^32^, ^33^ to realize highly efficient and cost‐effective multi‐junction devices. Currently, the perovskite based tandem efficiencies are still lower than the highest efficiency single junction bottom device,^34^, ^35^ primarily limited by the NIR‐ transparent perovskite top cells. Generally, efficient NIR‐transparent perovskite solar cells adopt a planar structure processed at low‐temperature (≈100 °C),^31^, ^36^, ^37^, ^38^ which usually delivers lower open circuit voltage *V*_OC_ (< 1.1 V) when compared to mesoporous structures employing high‐temperature (≈500 °C) processed TiO_2_.^11^, ^12^, ^13^ The loss in *V*_OC_ is particularly severe in planar devices using thick absorbers that are desired for current‐matching monolithic tandems. Novel concepts to improve *V*_OC_ while maintaining high short circuit current density (*J*_SC_) in NIR‐transparent planar perovskite solar cells are essential to achieve over 30% tandem efficiency in combination with the well‐established c‐Si or CIGS solar cells.

Compositional grading, which creates additional electric force induced by bandgap grading to assist the drift of photogenerated electrons and holes to respective contacts to be collected, has been designed to improve charge collection and suppress interface recombination in various state‐of‐the‐art solar cells, including CIGS,^39^ CdTe,^40^ CZTS,^41^ quantum dots,^42^ and GaAs.^43^ A similar concept has long been widely employed in silicon solar technology using chemical and/or field effect passivation enabled by tunneling insulator or back surface field enabled by heavy doping.^34^ The importance of graded absorber in improving *V*_OC_ and overall efficiency has been recognized in opaque perovskite solar cells very recently. Wu et al. reported a perovskite‐fullerene graded heterojunction to simultaneous improve *V*_OC_, *J*_SC_, and fill factor (FF), which yield 18.2% efficient perovskite cells with >1 cm^2^ area.^44^ Later, Ergen et al. designed a unique perovskite solar cell structure comprising wide bandgap MAPbI_3−_ *_x_*Br*_x_* and low bandgap MASnI_3_ double absorbers to obtain ultrahigh *J*_SC_ of 45 mA cm^−2^ and efficiency of 21.7%.^45^ The functionality of this double absorber architecture heavily relies on monolayer hexagonal boron nitride and graphene aerogel that require sophisticated processing. Most recently, Cho et al. achieved a thin composition gradient at the rear interface between the perovskite and hole transporting layer by constructing an additional FAPbBr_3−_ *_x_*I*_x_* on top of the primary (FAPbI_3_)_0.85_(MAPbBr_3_)_0.15_ film.^46^ This additional layer acted as an efficient electron blocking layer and resulted in an improved *V*_OC_ of 1.16 V (compared to 1.10 V in device without composition gradient) and efficiency of 21.3%. Importantly, a continuous Br concentration gradient along the whole perovskite absorber has been realized via a fume substitution doping method using HBr; however, the Br gradient eventually converted into a homogenous mixed halide layer through an ion migration based intermixing process.^47^ Despite various approaches to design graded perovskite absorbers, challenges remain in achieving stable and controllable grading from solution processing. Besides improved power conversion efficiency, the long‐term (>1000 h) stability of devices employing compositionally graded absorbers is yet to be demonstrated, and this is an essential factor determining the economic viability of the NIR‐transparent perovskite single junction and perovskite based tandem photovoltaics. It is very challenging to achieve improved efficiency while maintaining long‐term operational stability under continuous illumination, especially at temperature close to field condition where 60–80 °C can be easily reached.

Here we report a facile partial ion‐exchange (PIE) approach to fabricate compositionally graded perovskite absorbers, which simultaneous improves photovoltaic performance and operational stability in NIR‐transparent perovskite solar cells. The compositional grading is introduced by a simple chemical treatment, where organic bromide (MABr or FABr) solution is spin coated on primary absorbers (MAPbI_3_ or FAPbI_3_) to induce partial halide ion‐exchange. The resulting Br concentration gradient over the entire thickness induces a bandgap grading, which facilitates charge collection and reduces interface recombination. Consequently, we achieved improved *V*_OC_ of 1.116 V and steady‐state efficiency of 16.8% in NIR‐transparent perovskite solar cells with graded MAPbI_3−_ *_x_*Br*_x_* absorber layer. Moreover, non‐encapsulated graded perovskite devices demonstrate superior operational stability under long‐term heat/light soaking at maximum power point condition, retaining over 93% of its initial efficiency after 1000 h at 60 °C. In combination with Cu(In,Ga)Se_2_ solar cells, we obtain 22.7% efficiency in 4‐terminal tandem configuration with an absolute 3.3% improvement compared to the highest single junction bottom cell (19.4% CIGS).

## 2 Results and Discussion

### 2.1 Compositionally Graded Absorber via Partial Ion‐Exchange

**Figure 1:**
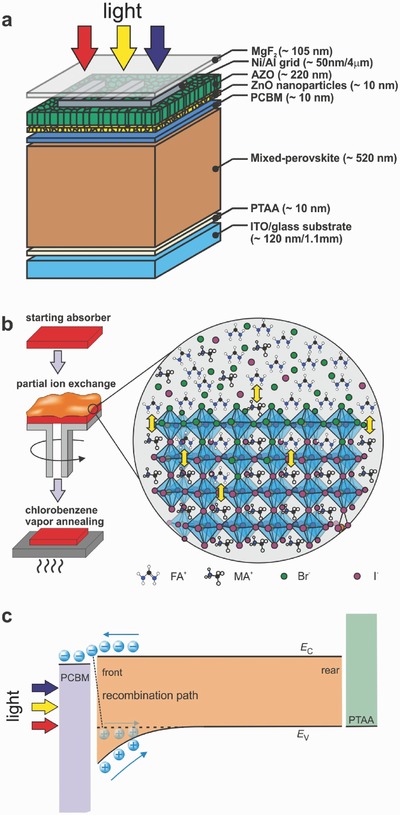
Compositionally graded perovskite absorber through partial ion‐exchange. a) Schematic device structure of NIR‐transparent perovskite solar cells (not to scale). The arrows represent the light illumination direction. b) Illustration of compositionally graded mixed‐cation lead mix‐halide perovskite absorbers prepared by partial ion‐exchange reaction. c) Schematics of the band diagram of the graded MAPbI_3−_ *_x_*Br*_x_* absorber. *E*_C_ and *E*_V_ represent the energetic positions of the conduction band minimum and the valence band maximum, respectively. Front and rear refer to perovskite/PCBM and perovskite/PTAA interface, respectively. The arrows indicate the light illumination direction.

**Figure** **1**a displays a schematic device structure of NIR‐transparent perovskite solar cells investigated in this study. We employed a substrate configuration with a planar stack of glass substrate/indium tin oxide (ITO)/poly[bis(4‐phenyl)(2,4,6‐trimethylphenyl)amine] (PTAA)/perovskite absorber (≈520 nm)/phenyl‐C61‐butyric acid methyl ester (PCBM)/ZnO nanoparticles/aluminum doped zinc oxide (AZO)/Ni‐Al grid/MgF_2_ antireflection coating. In Figure 1b, the compositionally graded perovskite absorber was prepared by PIE reaction as illustrated in MAPbI_3−_ *_x_*Br*_x_*. Essentially, the PIE reaction involves three different stages: (i) the starting absorber MAPbI_3_ was prepared by hybrid thermal evaporation/spin coating method;^28^, ^48^ (ii) then the as‐prepared absorber was subjected to a post‐deposition treatment by spin coating MABr solution in isopropanol to induce halide ion‐exchange at room temperature; (iii) finally the MABr‐treated film was thermally annealed under chlorobenzene vapor atmosphere to facilitate ions diffusion and redistribution, and to promote the crystal growth.^29^ For non‐graded absorbers, chlorobenzene vapor assisted thermal annealing was also applied to obtain large grain size.^29^ The PIE method can also be applied to FAPbI_3_ based graded absorbers when FAPbI_3_ and FABr (or MABr) are used as starting absorber and exchange precursor, respectively, and the results will be presented in the supplementary information. More details regarding fabrication processing of solar cells can be found in the Experimental section.

The incorporation of Br via partial halide ion‐exchange at the front interface and subsequent diffusion toward the rear interface is expected to form compositionally graded layer comprising a Br concentration that continuously decreases over the whole thickness of the perovskite absorber. In mixed halide perovskites, the valence band and conduction band are dominated by halide *p* orbitals and Pb 6p orbitals, respectively.^49^ Density functional calculations predicted that the valence band maximum (VBM) downshift with increasing Br content in MAPbI_3−_ *_x_*Br*_x_*, while the conduction band minimum (CBM) does not show significant change with Br composition variation.^50^ This prediction was experimentally verified in MAPb(I_1−_ *_x_*Br*_x_*)_3_ (0 ≤ *x* < 0.67) films deposited by co‐evaporation.^51^ These results demonstrated the easy tunability of VBM of MAPbI_3−_ *_x_*Br*_x_* by varying Br composition. Therefore, here we design a compositionally graded MAPbI_3−_ *_x_*Br*_x_* absorber via PIE to facilitate photogenerated carrier collection and suppress recombination loss, and the schematic band diagram is shown in Figure 1c. As light illuminated from PCBM/Perovskite interface, the front part of perovskite absorber corresponds to the highest generation region of photogenerated carriers in the solar cell. The recombination of electrons and holes at PCBM/perovskite interface is one of the recombination pathways that lower the *V*_OC_ and *J*_SC_, and the recombination rate is influenced by the concentration of both electrons and holes. Compared to nongraded cell, the Br compositional grading will introduce bandgap grading which assists the drift of the holes away from the front interface, i.e. the concentration of holes available directly at the interface is reduced. This effect is particularly pronounced under operation condition at maximum power point (MPP). In addition, the reduced hole concentration at front interface could suppress the interface recombination, leading to improved open‐circuit voltage (*V*_OC_) and *J*_SC_.

### 2.2 Photovoltaic Performance

**Figure 2:**
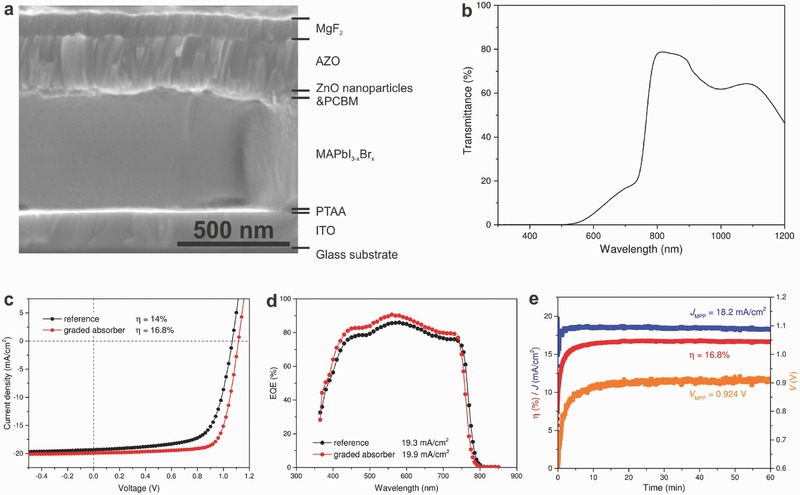
Photovoltaic performance of graded MAPbI_3−_ *_x_*Br*_x_* perovskite solar cells. a) The cross‐sectional SEM image and b) transmittance of graded MAPbI_3−_ *_x_*Br*_x_* perovskite solar cell. c) The *J*–*V* curves and d) EQE spectra of substrate configuration NIR‐transparent perovskite solar cells. The graded perovskite absorber was prepared by spin coating MABr (2.5 mg mL^−1^ in isopropanol) solution on MAPbI_3_. The MAPbI_3_ reference was also presented for comparison. e) The maximum power point measurement (MPP) of the best NIR‐transparent device. The cells were characterized in ambient air with 50% relative humidity.

**Figure** **2**a shows the cross‐sectional scanning electron microscopy (SEM) image of the graded MAPbI_3−_ *_x_*Br*_x_* perovskite solar cell. The 520 nm thick absorber exhibits flat and compact morphology with large and monolithic grown grains, which are believed to have beneficial effects on suppressed defect state and enhanced carriers transport properties as well as enhanced device stability.^52^ As both front and rear contacts are transparent conducting oxides, the perovskite solar cell exhibits high transmittance (Figure 2b) in the near‐infrared region, which makes it suitable for tandem application with low‐bandgap thin film CIGS solar cells. Figure 2c presents the photovoltaic performance of an optimized NIR‐transparent perovskite solar cell with graded MAPbI_3−_ *_x_*Br*_x_* absorber. The photovoltaic performance of a reference device without grading was also shown for comparison. The devices are measured in substrate configuration (device illuminated from the film side) under standard test conditions (STC: 25 °C, simulated AM1.5G, 100 mW cm^−2^).^29^ After incorporating the grading by PIE with MABr solution (concentration of 2.5 mg mL^−1^), the device performance improved substantially from 14% to 16.8%, with concurrent improvement in all photovoltaic parameters, particularly the *V*_OC_ and FF. We achieved highest power conversion efficiency (η) of 16.8%, with a *V*_OC_ of 1.116 V, a *J*_SC_ of 19.9 mA cm^−2^, and a FF of 75.7%. As shown in Figure S1 in the Supporting Information, the cell shows negligible *J*–*V* hysteresis when scanned in both forward (−0.75 to 1.2 V) and backward (1.2 to −0.75 V) direction with 20 mV step size and 1 s delay time. It is to be noted that the *J*_SC_ values are obtained from integrated external quantum efficiency (EQE) spectra as shown in Figure 2d, from which we can see that the EQE shifted upward in the whole response range and the absorption edge shifted toward lower wavelengths. The blue shift of the absorption edge translates to an increased bandgap from 1.60 to 1.62 eV after PIE as shown in Figure S2 in the Supporting Information. Despite the increased bandgap, the increased EQE response suggests an improved charge collection enabled by compositional grading. In addition, the comparison of dark *J*–*V* curves is shown in Figure S3 in the Supporting Information, from which the reverse saturation current density *J*_0_ can be extracted by fitting the *J*–*V* curves with one‐diode model. The *J*_0_ usually corresponds to recombination of electrons and holes.^53^ The graded device (MAPbI_3_:MABr (2.5 mg mL^−1^)) showed lower *J*_0_ than reference device (MAPbI_3_), indicating a suppressed recombination after incorporating Br composition grading. To further evaluate the actual efficiency at operational conditions, we kept the best performing cell at the MPP under continuous illumination under STC. The photovoltaic parameters at MPP as a function of time are plotted in Figure 2e. The cell reached a steady‐state η of 16.8%, *J*_MPP_ of 18.2 mA cm^−2^, and *V*_MPP_ of 0.924 V after over 60 min of MPP operation in ambient air, in good agreement with the *J*–*V* characterizations. We note that the devices studied here show reversible light soaking effect as shown in Figure S4 in the Supporting Information.^29^ The statistics of 15 devices with graded absorbers (Figure S5, Supporting Information) indicates good reproducibility with an average efficiency of 15.6%. We further applied the PIE process on solar cells grown on 5 cm × 5 cm substrate, and achieved a steady state efficiency of 15.7% for a NIR‐transparent device with 0.561 cm^2^ cell area, as shown in Figure S6 in the Supporting Information. The thermally evaporated PbI_2_ layer in hybrid vapor/solution method ensures a homogeneous deposition of perovskite over large area, showing potential to scale up to even larger substrate size for NIR‐transparent perovskite mini‐modules and tandem mini‐module devices.

By varying the MABr concentration prior to the PIE process, the amount of Br incorporated into the absorber and the extent of grading could be controlled. Figure S7 in the Supporting Information presents the *J*–*V* curves and the EQE spectra of the NIR‐transparent devices with MAPbI_3−_ *_x_*Br*_x_* graded absorber layers fabricated with various concentration of MABr (0 to 7.5 mg mL^−1^). The devices subjected to halide ion‐exchange all showed enhanced performance, and a highest *V*_OC_ of 1.119 V was achieved at a MABr concentration of 5 mg mL^−1^ in the spin coating solution. Further optimization is needed to improve the FF and obtain higher efficiency. From the enlarged EQE spectra in Figure S7b in the Supporting Information, it is observed that the absorption edge shows a systematic blue shift with increasing MABr concentration. This confirms the amount of Br incorporated into the absorber correlates to the initial MABr precursor concentration prior ion‐exchange. In addition to the organic halide precursor concentration, loading time (the time between spreading the precursor solution and the onset of spinning), precursor and substrate temperature offer additionally parameters for further optimization to obtain the desired graded profile. The ion‐exchange reaction between the halide ions was also substantiated by the shift of the perovskite (110) main peak toward higher angles with increasing MABr concentration as shown in the X‐ray diffraction (XRD) patterns (Figure S8, Supporting Information) of the full devices. We note that a residual PbI_2_ peak was detected in the XRD patterns of high efficiency cells. Considering the compact morphology of the thermally evaporated PbI_2_ film (Figure S9, Supporting Information), the residual PbI_2_ is most likely located at the rear perovskite/PTAA interface since perovskite is formed from top to bottom. The beneficial effect of residual PbI_2_ has been reported in our previous work and increasing evidences have shown that an excess amount of PbI_2_ is necessary to obtain highly efficient perovskite devices regardless of the device architecture and fabrication methods.^13^, ^28^, ^29^, ^54^, ^55^ The underlying mechanism is beyond the scope of this work and further comprehensive electronic, spectroscopy, microstructural, and photovoltaic characterizations are needed to fully understand the mechanism.

To demonstrate the versatility of the compositional grading strategy in improving *V*_OC_ and efficiency, we applied the PIE approach to other perovskite systems (starting absorber:exchanged solution), including FAPbI_3_:FABr and FAPbI_3_:MABr. The morphology and composition as well as photovoltaic performance are shown in Figure S10–15 in the Supporting Information. We observed that the *V*_OC_ and device performance increased considerably after organic cation and/or halide anion exchange in FAPbI_3_:FABr and FAPbI_3_:MABr. It is important to note that further optimization of processing parameters and charge selective layers would lead to higher performance for FAPbI_3_ based devices. In addition to the enhanced efficiency, the operational stability of graded bandgap perovskite devices improved significantly due to the incorporation of Br. For instance, a non‐encapsulated FAPbI_3_: FABr (7.5 mg mL^−1^) device remained stable for 5 h operation at MPP at a temperature of 80 °C in ambient air with 50% relative humidity (Figure S12, Supporting Information). Long‐term (1000 h) light soaking under continuous illumination at various temperatures will be discussed in more detail later. These results demonstrate the benefits of compositional grading as a promising strategy to enhance the device performance and operational stability of NIR‐transparent perovskite solar cells.

### 2.3 Compositional Grading Characterization

**Figure 3:**
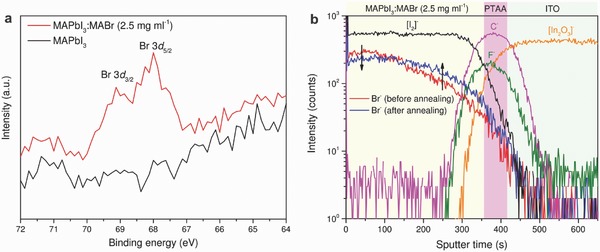
Compositional grading. a) The high resolution X‐ray photoelectron spectroscopy (XPS) data of Br 3d core‐level spectra for PIE prepared absorber (MAPbI_3_:MABr 2.5 mg mL^−1^) and reference one (bare MAPbI_3_). Both absorbers are prepared according to same protocol for solar cells fabrication. b) The ToF‐SIMS depth profile of graded absorber after chlorobenzene vapor assisted thermal annealing (60 min at 100 °C). The Br depth profile in absorber prior to annealing is plotted for comparison.

To verify that the PIE reaction can form compositionally graded absorber, we elucidate the compositional and morphological changes during the PIE process. We first quantify the amount of Br actually incorporated into the absorber in respect of the nominal MABr concentration by performing high resolution X‐ray photoelectron spectroscopy (XPS) analysis on the MAPbI_3_:MABr (2.5 mg mL^−1^) and the control (bare MAPbI_3_) absorbers. **Figure** **3**a presents the Br 3d spectra, and the relevant characteristic elements are shown in Figure S16 in the Supporting Information. The O 1s peak at around 532 eV is negligible in both samples, indicating minimum contamination during the transfer of samples from glovebox to the XPS chamber. The Br peaks at 69.1 eV (Br 3d_3/2_) and 68 eV (Br 3d_5/2_) binding energies are clearly detected (Figure 3a). The calculated Br concentration is around 2 at% on the surface for MAPbI_3_:MABr (2.5 mg mL^−1^) sample. We note that quantification of Br in the whole absorber is difficult due the to the Br grading. It is found that the XPS peak position and intensity of I, C, N, Pb signals did not change appreciably after halide anion exchange, suggesting no significant chemical structure changes after Br incorporation.

Having determined the actual Br concentration on the surface of the absorber, we proceed to reveal the distribution of Br throughout the absorber by time‐of‐flight secondary ion mass spectrometry (ToF‐SIMS) elemental depth profiling. Three absorbers corresponding to different stages illustrated in Figure 1b were studied here to reveal the Br evolution during PIE processing. The ToF‐SIMS depth profiles and the SEM images are shown in Figure S17 in the Supporting Information. After spin coating of the MABr solution, the as‐spun sample shows a very similar morphology as the starting MAPbI_3_ absorber in low‐magnification SEM image. Higher magnification SEM images (Figure S18, Supporting Information) reveals nanostructured morphology on the rough surface of as‐spun sample, indicating strong chemical reaction on the surface and along grain boundaries. After chlorobenzene vapor assisted thermal annealing, the grains size increased considerably and the surface became smooth (Figure S17c, Supporting Information). The elemental depth profiles in Figure S17g,h in the Supporting Information confirm the incorporation of Br into the absorber immediately after spin coating. Moreover, a strong Br gradient is clearly detected, which supports the morphology observation (Figure S18, Supporting Information) that ion exchange mainly occurred at the surface of the absorber during spin coating. After chlorobenzene vapor assisted thermal annealing, the Br intensity decreased on surface and increased in the absorber (Figure S17i, Supporting Information), indicating a further diffusion of Br ions from surface toward bottom perovskite/PTAA interface. This change is clearly visualized in Figure 3b, where the depth profiles of Br prior and after vapor annealing are plotted together. Despite the redistribution of Br ions, the Br gradient remained. As shown in depth profile in Figure S19 in the Supporting Information, the Br gradient was not detected when mixed‐halide perovskite absorber was prepared by spin coating blended MAI/MABr precursor solution onto PbI_2_ film. This confirms that the Br gradient was introduced by the here described PIE process. The Br graded absorber is beneficial for the collection of photogenerated carriers due to the additional electric force to assist charge drifting.^47^, ^56^, ^57^ The increased collection of carriers and reduced recombination at the front interface are believed to contribute to the improved *V*_OC_ and enhanced EQE in the whole absorption range.

### 2.4 Operational Stability at Various Temperatures

**Figure 4:**
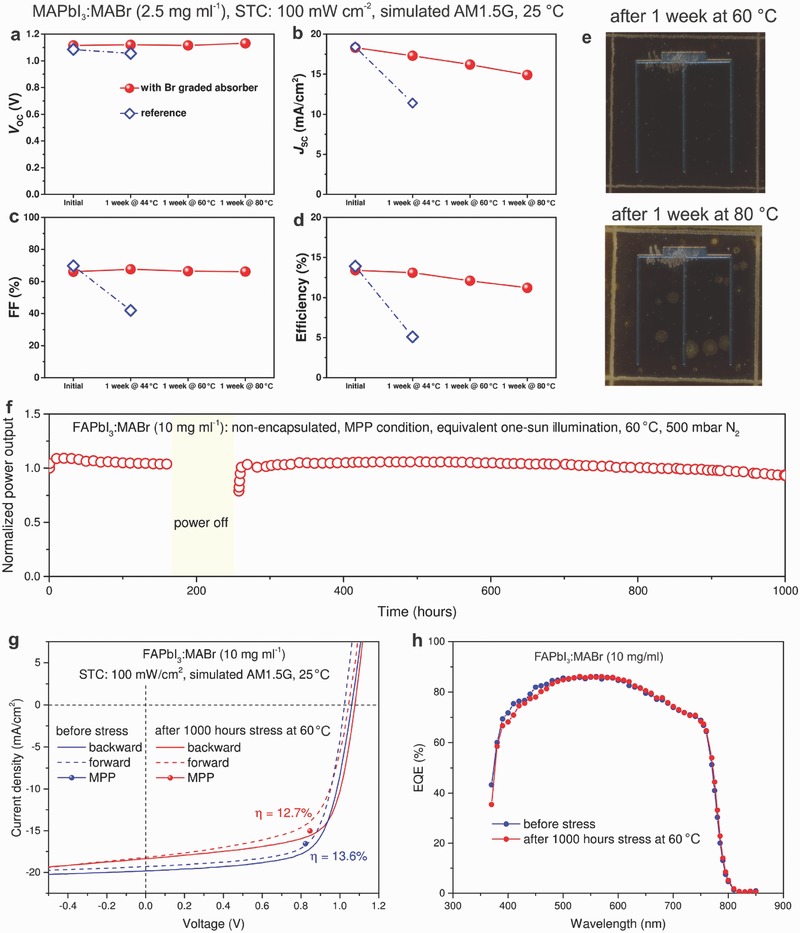
Operational stability at elevated temperature. a) The *V*_OC_, b) *J*_SC_, c) FF, and d) efficiency evolution of graded perovskite solar cells measured under STC after stressing at different conditions. Non‐encapsulated graded (MAPbI_3_:MABr (2.5 mg mL^−1^)) and reference (MAPbI_3_) devices were kept at MPP condition under continuous equivalent 1 sun illumination at various temperature for 1 week. White light emitting diodes (LEDs) array was employed as light source and temperature was controlled and monitored by temperature sensor near the device. The devices were stressed under full area illumination in 500 mbar N_2_ atmospheres. e) The photographs of graded perovskite (MAPbI_3_:MABr (2.5 mg mL^−1^)) cell after 1 week stress at 60 and 80 °C. f) The normalized efficiency of graded perovskite cells using FAPbI_3_:MABr (10 mg mL^−1^) absorbers. Power supply was unintentionally off during the measurement. g) The *J*–*V* curves and h) EQE spectra of graded mixed cations and mixed‐halide perovskite (FAPbI_3_:MABr (10 mg mL^−1^)) solar cells measured before and after 1000 h stress at 60 °C under STC.

Previous work on graded perovskite solar cells mainly focused on optimizing the power conversion efficiency, but the performance stability under operational condition at temperature close to field condition have not been reported. Encouragingly, here we found that the incorporation of a Br gradient via PIE not only improved photovoltaic performance but also substantially enhanced operational stability under continuous illumination at temperatures up to 80 °C. We compared the operational stability of the reference (MAPbI_3_) and graded (MAPbI_3_:MABr (2.5 mg mL^−1^)) NIR‐transparent perovskite solar cells by operating them at MPP condition under continuous equivalent 1 sun illumination. The non‐encapsulated cells were stressed under full area illumination under 500 mbar N_2_ atmosphere. A white light emitting diode (LED) array was employed as the light source. After continuous MPP operation for 1 week under 1 sun illumination (no temperature control of the cell was targeted in this experiment; the temperature rises to 44 °C within several hours without intentional heating), the device was immediately taken out of the stress chamber to conduct *J*–*V* characterization under STC. The *J*–*V* curves of reference and graded devices measured under STC are shown in Figures S20 and S21 in the Supporting Information, and the photovoltaic parameters as function of stress condition are summarized in **Figure** **4**a–d. The reference sample lost over 60% of its initial efficiency (Figure 4a–d) due to the decrease in all photovoltaic parameters, particularly the FF and *J*_SC_; while for the graded device, *V*_OC_ increased ≈0.8% (+9 mV), *J*_SC_ decreased ≈5.5% (−1 mA cm^−2^), and the FF increased ≈2.6% (+1.7%), resulting in only ≈2.2% relative decrease in overall efficiency. To further assess the operational stability at elevated temperature close to field condition, the graded device was further stressed at 60 and 80 °C consecutively each for 7 d under continuous 1 sun illumination at MPP. As seen from Figure 4a–d, the device retained above 92% of its initial performance after each stress cycle. In our previous work, MAPbI_3_ based device already lost over 30% of its initial efficiency only after 60 h light soaking at 65 °C.^29^ This result demonstrates that Br‐grading can significantly improve the device operation stability. It is clearly observed from Figure 4a–d that the degradation in graded perovskite device was dominated by a decrease in the *J*_SC_. The *V*_OC_ and FF remained almost unchanged after 1 week stressing at each temperature (in total 500 h), indicating very good photo‐ and thermal‐stability of the functional layers and junctions. Considering the full area illumination on non‐encapsulated device, the *J*_SC_ loss probably mainly comes from the lack of edge seal and decomposition of perovskite induced by impurities (probably from processing steps or low‐grade antireflection coating). The photograph of a graded device (Figure 4e) after stress at 60 and 80 °C shows that the scribe lines became wider and yellowish. Many yellow spots appeared from original white spots after stressing at 80 °C for 1 week, suggesting decomposition from perovskite to PbI_2_. The decomposition of perovskite could be accelerated by heat and under‐pressure atmosphere.^58^, ^59^

Although the stability improved considerably in graded MAPbI_3−_ *_x_*Br*_x_* solar cells, concerns on phase segregation and homogenization of composition grading remains. To further assess the stability of graded perovskite solar cells, we performed long‐term (1000 h) heat/light soaking test on graded FAPbI_3_:MABr (10 mg mL^−1^) perovskite solar cells. Figure 4f shows the normalized efficiency of non‐encapsulated graded FAPbI_3_:MABr (10 mg mL^−1^) devices operating at MPP condition under equivalent 1 sun illumination at 60 °C. The graded FAPbI_3_:MABr (10 mg mL^−1^) perovskite device retained over 93% of its initial efficiency after 1000 h heat/light soaking test, while nongraded FAPbI_3_ only remained 75% of its initial efficiency under the same conditions (Figure S22, Supporting Information). The *V*_OC_, *J*_SC_, FF, and stress condition as a function of time for graded FAPbI_3_:MABr (10 mg mL^−1^) perovskite device were shown in Figure S23 in the Supporting Information, from which we can see that the efficiency loss primarily comes from decreased *J*_SC_ and FF, while *V*_OC_ was gradually increased to over 1 V during the heat/light soaking test. The same trend was also observed when comparing the photovoltaic performance of graded device measured before and after heat/light soaking under STC as shown in Figure 4g. The absorption onset in the EQE spectra (Figure 4h) measured before and after 1000 h heat/light soaking remains almost unchanged, suggesting that the composition grading is very stable under the tested condition. Phase segregation and homogenization of composition grading are not observed under current stress condition as either of them would deteriorate the performance drastically and shift the absorption edge. We note that the *J*_SC_ extracted from *J*–*V* scans is lower than the *J*_SC_ integrated from EQE spectrum after the heat light soaking, suggesting the decomposition of perovskite absorber near the scribe line and metal grid finger (Figure S24, Supporting Information) that reduce the active area. It is concluded from the above stability investigations that introducing small amount of Br significantly improves the operational stability in both MAPbI_3_ and FAPbI_3_ cases. The improved operational stability might be mainly ascribed to a more stable crystal structure induced by Br incorporation.^60^

The aim and consequences of MABr treatment is substantially different from previous work where low‐concentration MABr solution is employed to induce Ostwald ripening and ion‐exchange is detrimental to device performance and should be avoided.^61^ In our work the partial halide ion‐exchange is essential for designing and implementing the compositional grading. Compared to previously reported methods to design graded perovskite absorbers,^45^, ^46^, ^47^ the here described PIE approach have several distinct advantages: Firstly, the PIE reaction offers an facile approach to design graded absorber via simple postdeposition treatment. Instead of double absorbers used in previous works,^45^, ^46^ a single absorber with graded composition is achieved by PIE reaction and no complicated diffusion barrier nor control of residual PbI_2_ is required. Secondly, the graded perovskite solar cells prepared by PIE demonstrate significantly improved operational stability under continuous light soaking at temperature close to field condition. In contrary, the device using double layer structure have poor stability under continuous light illumination.^45^ Thirdly, the concept of using PIE to achieve compositional grading could potentially be employed to design double grading which is widely employed in highly efficient CIGS thin film solar cells.^62^

### 2.5 Perovskite‐CIGS Thin Film Tandem Solar Cells

**Figure 5:**
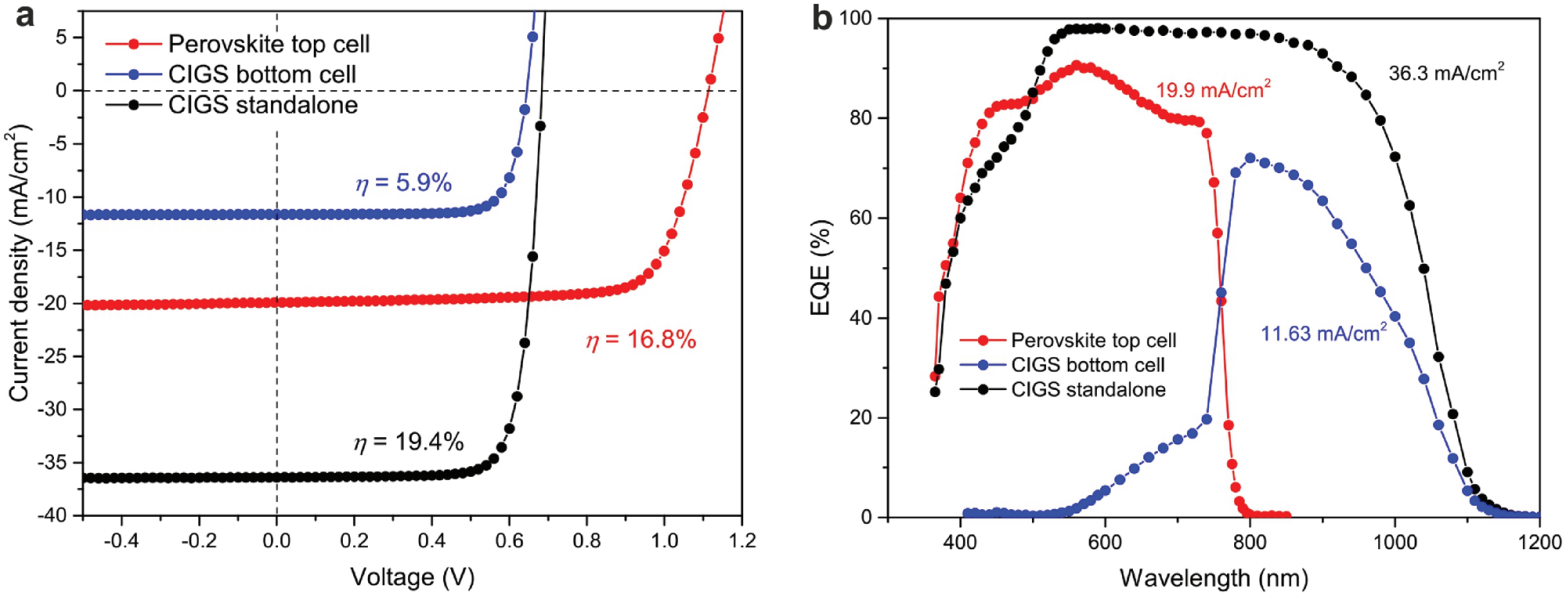
Perovskite‐CIGS thin‐film tandem solar cells. a) The *J*–*V* curves and b) EQE spectra of the perovskite‐CIGS thin‐film tandem solar cells in 4‐terminal configuration.

Finally, with such efficient and stable NIR‐transparent perovskite solar cells, we demonstrated mechanically stacked perovskite‐CIGS devices in 4‐terminal tandem configuration, where a perovskite top cell and CIGS bottom cell are individually processed and mechanically stacked together.^26^ The 4‐terminal tandem configuration enables us to optimize the top cell and bottom cell separately. The CIGS fabrication process was modified from previous work by implementing RbF postdeposition treatment.^63^, ^64^ The *J*–*V* curves and EQE spectra of the tandem subcells are displayed in **Figure** **5**, and the corresponding photovoltaic parameters are summarized in **Table** **1**. We started with a 16.8% perovskite top cell, which absorbs the visible light and transmits the near‐infrared light (as well as part of visible light due to incomplete absorption in perovskite top cell) into bottom CIGS cell. The standalone CIGS cell has an initial efficiency of 19.4%, which contributed 5.9% when operating as bottom subcell in 4‐terminal tandem configuration after stacking perovskite subcell on top of it. The mechanism of efficiency improvement by tandem concept is schematically illustrated in Figure S25 in the Supporting Information. As the top and bottom subcells operated at the respective maximum power point, the addition of top and bottom subcells efficiencies in yields 22.7% efficiency in 4‐terminal tandem configuration. This is the best value reported so far for perovskite‐CIGS thin film tandem devices, which is comparable to the current record efficiency of 22.6% for CIGS single junction grown at high temperature of ≈600 °C.^35^ Importantly, we achieved an absolute efficiency gain of 3.3% compared to the highest efficient subcell (19.4% CIGS in this case), this value is also comparable to the best‐performing perovskite/Si and all‐perovskite tandem solar cells.^22^, ^23^, ^25^, ^33^

**Table 1:** Photovoltaic parameters of the thin‐film perovskite‐CIGS solar cells in 4‐terminal tandem configuration. An absolute efficiency gain of 3.3% is achieved compared to the highest efficient subcell

Solar cell	*V*_OC_ [V]	*J*_SC_ [mA cm^−2^]	FF [%]	η [%]	MPP [%]	Cell area [cm^2^]	Absolute gain
Perovskite top cell	1.116	19.9	75.7	16.8	16.8	0.273	
CIGS (standalone)	0.684	36.4	78.2	19.4	19.4	0.213	
CIGS bottom cell	0.645	11.6	78.1	5.9	5.9	0.213	
Perovskite‐CIGS 4‐terminal tandem					22.7	0.213	3.3%

## 3 Conclusions

In conclusion, we have developed a facile partial ion‐exchange strategy to fabricate compositionally graded perovskite absorber to simultaneously improve the efficiency and operational stability in NIR‐transparent perovskite solar cells. Spin coating of organic bromide (MABr or FABr) solution onto starting MAPbI_3_ or FAPbI_3_ absorber induced halide ion‐exchange and subsequent ions diffusion, which results in a perovskite absorber with a Br composition gradient as verified by ToF‐SIMS element depth profiling. Incorporating compositional grading improved charge collection and suppressed interface recombination, enabling us to achieve improved *V*_OC_ and *J*_SC_, and a steady state efficiency of 16.8% in NIR‐transparent perovskite solar cells with thick absorber (520 nm). In addition to improved efficiency, non‐encapsulated NIR‐transparent perovskite device with graded absorber retained over 93% of its initial efficiency after 1000 h operation at MPP condition at 60 °C under equivalent 1 sun illumination. When mechanically stacked on CIGS solar cells, we demonstrated 22.7% efficiency in 4‐terminal tandem configuration with 3.3% absolute efficiency gain compared to the highest single junction cell (19.4% CIGS). The PIE approach offers a viable way to tailor the composition and morphology of the mixed‐perovskite absorbers which is not easily accessible by other methods, and our results provide new direction in exploring compositional grading via partial ion‐exchange to perovskite solar cells.

## 4 Experimental Section

*Perovskite Solar Cells Fabrication*: Perovskite solar cells were grown on commercial ITO coated glass (sheet resistance: 8 Ω sq.^−1^, Zhuhai Kaivo Optoelectronics, P. R. China). The ITO glass was washed by hand first and then subjected to soap and deionized water sonification bath at 85 °C each for 15 min. The ITO glasses were then dried by compressed nitrogen gun and used for solar cells processing without additional ozone treatment. The hole transporting layer was prepared by spin coating 30 µL of PTAA (Sigma‐Aldrich) solution (5 mg mL^−1^ in toluene doped with 1 wt% F4‐TCNQ (97%, Sigma‐Aldrich)) at 6000 r.p.m. for 45 s on 2.5 × 2.5 cm^2^ substrate, followed by thermal annealing at 100 °C for 10 min. Afterward, around 200 nm PbI_2_ (ultradry, 99.999%, Alfa Aesar) compact film was thermally evaporated on rotating PTAA/ITO/glass without intentional heating. The deposition rate was controlled within 1–1.5 Å s^−1^, and the deposition pressure was between 3 and 6 × 10^−8^ mbar. After the PbI_2_ deposition, samples were transferred into glovebox for further processing. The mixed‐perovskite layers were formed by partial ion exchange reaction which contains mainly three stages. Firstly, the starting absorber, such as MAPbI_3_ (or FAPbI_3_) were prepared as follows: 300 µL of MAI (or FAI) solution (65 mg mL^−1^ in isopropanol) was first spread onto PbI_2_ surface, and then immediately started the rotation at 6000 r.p.m. for 45 s. The as‐deposited films were annealed at 100 (or 150 °C) for 10 min for MAPbI_3_ (or for FAPbI_3_). The starting absorber (MAPbI_3_ or FAPbI_3_) were subjected to spin coating (6000 r.p.m. for 45 s) of organic bromide (MABr, FABr) to induce the ion‐exchange. Afterward, the absorbers were annealed at 100 °C for 1 h under chlorobenzene vapor atmosphere. For electron transporting layer, 30 µL PCBM (PC_61_BM, 99.5%, Solenne BV, Netherland) solution (20 mg mL^−1^ in chlorobenzene) was spin coated at 5000 r.p.m. for 45 s followed by 60 min annealing at 100 °C covered by petri dish with presence of 10 µL chlorobenzene. After cooling down, 30 µL undoped ZnO nanoparticles (2.7 wt% (crystalline ZnO dissolved in isopropanol), Sigma‐Aldrich) was spin coated on top of PCBM at 4000 r.p.m. for 45 s. The ZnO nanoparticles were dried at 100 °C for 60 s to evaporate the solvent. Finally, the samples were coated with ZnO:Al front contact by RF‐magnetron sputtering and Ni/Al (50 nm/4000 nm) metallic grid by e‐beam evaporation. Finally, all cells were covered with a 105 nm MgF_2_ antireflection coating deposited by e‐beam evaporation and single cells were defined by mechanical scribing down to ITO back contact.

*Deposition of Al:ZnO Front Contact*: Al:ZnO layers were deposited in a high vacuum sputtering system by RF‐magnetron sputtering of ceramic ZnO (containing 2 wt% Al_2_O_3_) target (99.995%, Materion). The deposition consisted of a 5 min deposition ramp up from 0.6 to 2.5 W cm^−2^ and followed by 3 × 3 min at 2.5 W cm^−2^ under 20 sccm Ar, and 0.29 sccm Ar/O_2_ (3 mol% O_2_). There was a 30 min waiting time during each step to minimize the temperature effect. The sheet resistance of as‐deposited film on glass was around 56 Ω sq.^−1^ measured by 4‐point probe method.

*ToF‐SIMS*: Depth profiling data were obtained with ToF‐SIMS 5 system from ION‐TOF. Bi_3_^+^ ions were used as primary ions and negative ions were detected. Sputtering was performed using Cs^+^ sputtering ions with 500 – 1000 eV ion energy, 20–50 nA ion current and a 500 × 500 µm^2^ raster size. An area of 100 × 100 µm^2^ was analyzed using Bi_3_^+^ ions with 25 keV ion energy.

*XPS*: The XPS measurements were performed in a Quantum2000 from Physical Electronics using a monochromatized Al Kα source (1,486.6 eV). The measurements were recorded in the fixed analyzer transmission mode, under an angle of emission of 45° and at an instruments base pressure of below 5 × 10^−9^ mbar. Spectra were recorded with a pass energy of *E*_p_  =  29.5 eV and an energy step size of Δ*E*  =  0.125 eV. Ar^+^ ions and electrons were used to compensate possible surface charging. The Br concentration was calculated by fitting the data and using the relative sensitivity factor provided by Multipack software.

*XRD*: XRD patterns were obtained in Bragg–Brentano geometry by using a X'Pert PRO θ–2θ scan (Cu‐K_α1_ radiation, λ = 1.5406 Å) from 10 to 60° (2θ) with a step interval of 0.0167°.

*SEM*: The cross‐sectional SEM images of the samples were investigated with a Hitachi S‐4800 Scanning Electron Microscope using 5 kV acceleration voltage. A thin layer (≈1 nm) of Pt was coated on top of the samples to avoid charging effects.

*UV–vis–NIR spectroscopy*: The total transmittance (*T*) and reflectance (*R*) spectra were acquired using a UV–vis–NIR spectrophotometer (Shimadzu UV‐3600) equipped with an integrating sphere.

*Solar Cells Characterization*: The current density–voltage characteristics of perovskite solar cells were measured using a ABA class solar simulator and Keithley 2400 source meter. The illumination intensity was calibrated to 1000 W m^−2^ using a certified single crystalline silicon solar cell. The substrate temperature was kept at 25 °C using Peltier element. The *J*–*V* measurements were performed in both forward (form −0.5 to 1.2 V) and backward (from 1.2 to −0.5 V) direction seperately without any pretreatment (e.g., holding at forward bias for certain time etc.). The step size was 20 mV and the scan velocity varied from18 to 190 mV s^−^^1^. The external quantum efficiency of the cells was measured with a lock‐in amplifier. The probing beam was generated by a chopped white source (900 W, halogen lamp, 260 Hz) and a dual grating monochromator. The beam size was adjusted to ensure that the illumination area was fully inside the cell area. The shading effect of metallic grid was taken into account by including middle grid line into the illuminated area. A certified single crystalline silicon solar cell was used as the reference cell. White light bias was applied during the measurment with a halogen bias lamp. A spectral missmatch correction was applied to the *J*–*V* measurements based on the EQE current. The *J*_SC_ value was obtained by integrating the EQE spectrum over the AM1.5G photon flux and used to calculate the power conversion efficiency of the solar cells. The steady‐state efficiency as a function of time was recorded using a maximum power point tracker, which adjustsed the applied voltage in order to reach the maximum power point (perturb and observe algotrithm). The starting voltage was set to be 0.1 V.

*4‐Terminal Tandem Measurements*: The efficiency of the device in 4‐terminal configuration was determined as the sum of the top cell efficiency determined as described above and the bottom cell efficiency measured as follows: The perovskite top cell was stacked on top of the CI(G)S bottom cell using a laser scribed insulating mask to separate the bottom cell from the top cell. The corresponding aperture area was defined by the mask openning and was 0.213 cm^2^ in all measurements shown here. The metallic grid in top cell was also taken into account during the characterization of bottom cells. The measurements of *J*–*V* and EQE were carried out with the methods described above while being illuminated through the complete semitransparent perovskite top cell (including metallic grid). A spectral correction was applied to the *J*–*V* measurements based on the EQE current.

## Conflict of Interest

The authors declare no conflict of interest.

## Supporting information

Supplementary
