# Supplementary material for: Compositionally Graded Absorber for Efficient and Stable Near‐Infrared‐Transparent Perovskite Solar Cells
Source: Adv Sci (Weinh). 2018 Jan 5;5(3):1700675. doi: 10.1002/advs.201700675 (PMC5867048; doi:10.1002/advs.201700675)
Supplement: Supplementary file 1 — Supplementary [file ADVS-5-1700675-s001.pdf]

Copyright WILEY-VCH Verlag GmbH & Co. KGaA, 69469 Weinheim, Germany, 2017.

Supporting Information

for *Adv. Sci.,* DOI: 10.1002/advs.201700675

Compositionally Graded Absorber for Efficient and Stable Near-Infrared-Transparent Perovskite Solar Cells

Fan Fu, Stefano Pisoni, Thomas P. Weiss, Thomas Feurer, Aneliia Wäckerlin, Peter Fuchs, Shiro Nishiwaki, Lukas Zortea, Ayodhya N. Tiwari, and Stephan Buecheler**

Copyright WILEY-VCH Verlag GmbH & Co. KGaA, 69469 Weinheim, Germany, 2016.

Supporting Information

Compositionally graded absorber for efficient and stable near-infrared-transparent perovskite solar cells

Fan Fu, Stefano Pisoni, Thomas P. Weiss, Thomas Feurer, Aneliia Wäckerlin, Peter Fuchs, Shiro Nishiwaki, Lukas Zortea, Ayodhya N. Tiwari, Stephan Buecheler**

Laboratory for Thin Films and Photovoltaics, Empa-Swiss Federal Laboratories for Materials Science and Technology, Ueberlandstrasse 129, CH-8600 Duebendorf, Switzerland
E-mail: Fan.Fu@empa.ch, Stephan.Buecheler@empa.ch

Keywords: NIR-transparent perovskite solar cells, partial ion-exchange, compositional grading, operational stability, tandem solar cell

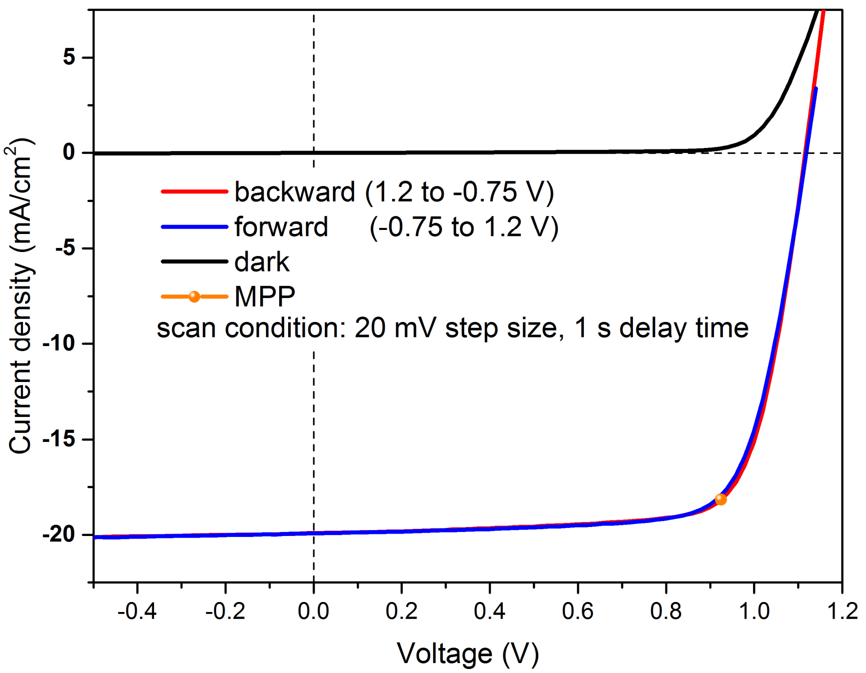

Figure S1. ***J-V*** **hysteresis of the best performing NIR-transparent graded perovskite solar cell.** The *J-V* scans were performed in both forward (-0.75 to 1.2 V) and backward (1.2 to -0.75 V) direction with 20 mV step size and 1 s delay time. The device shows negligible *J-V* hysteresis.

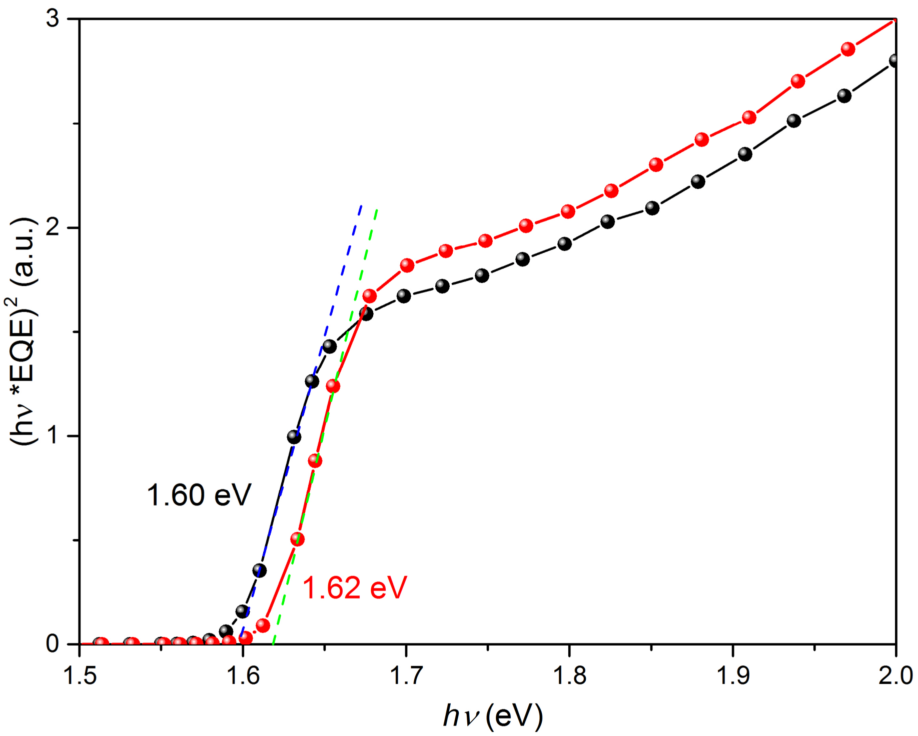

Figure S2. Band gap extracted from EQE spectra for reference ($MAPbI_3$) and graded absorbers ($MAPbI_3$: MABr (2.5 mg ml^{-1})). The bandgap increased from 1.60 eV to 1.62 eV after implementing compositional grading via PIE.

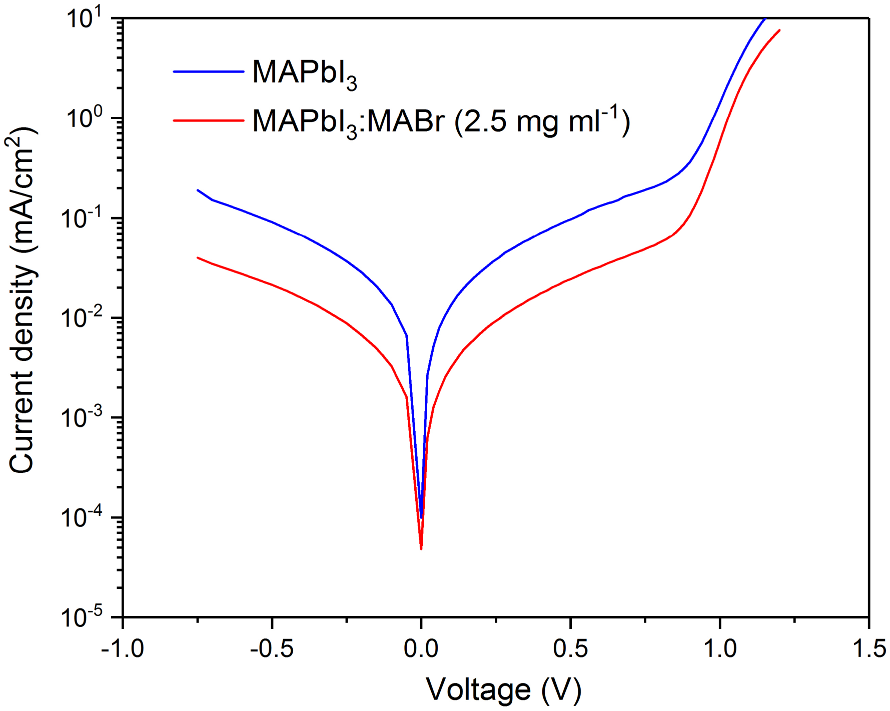

Figure S3. Dark *J-V* curves. The comparison of dark *J-V* curves of graded cell ($MAPbI_3$:MABr (2.5 mg ml^{-1})) and reference cell ($MAPbI_3$). Fitting the dark *J-V* curves with one diode model, the ideality factor *n* and reverse saturation current density J_0 can be extracted. The parameter J_0 can be identified as the indicator for recombination between electrons and holes (A. Cuevas. Energy Procedia 55, 53 (2014)). The graded device (J_0 = 9.8 × 10^{-11} mA/cm^2) show lower J_0 than that of reference cell (J_0 = 3.7 × 10^{-9} mA/cm^2), suggesting a reduced recombination after incorporating Br compositional gradient.

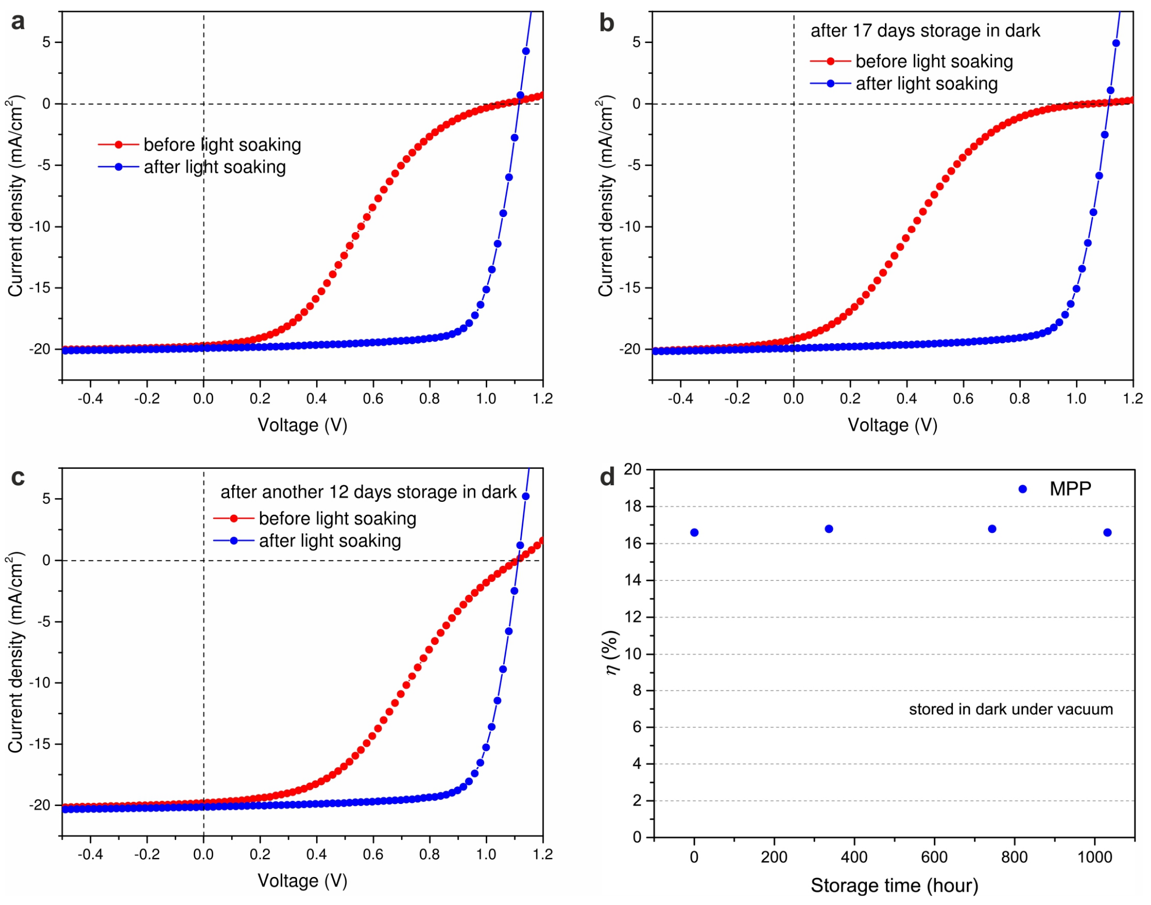

Figure S4. Reversible light soaking effect and shelf stability. a, **b**, **c**, The *J*-*V* curves of the best performing device measured after different storage time. When the cell was first exposed to light, the *J*-*V* curve exhibited S-shape with strong roll-over, mainly limiting the FF. The S-shape totally disappeared after continuous AM1.5G illumination for over 60 min in ambient air. When the device was stored in the dark, the device relaxed back to initial state and restored to the light-soaking state after illumination as shown in **b** and **c**. The light soaking treatment mainly affects the FF, while it has a negligible effect on J_{SC} and V_{OC}. **d**, The shelf stability of the cell stored in the dark over 1000 hours. Despite the reversible light soaking effect, the steady-state power conversion efficiency remained stable over 1000 hours storage in the vacuum oven.

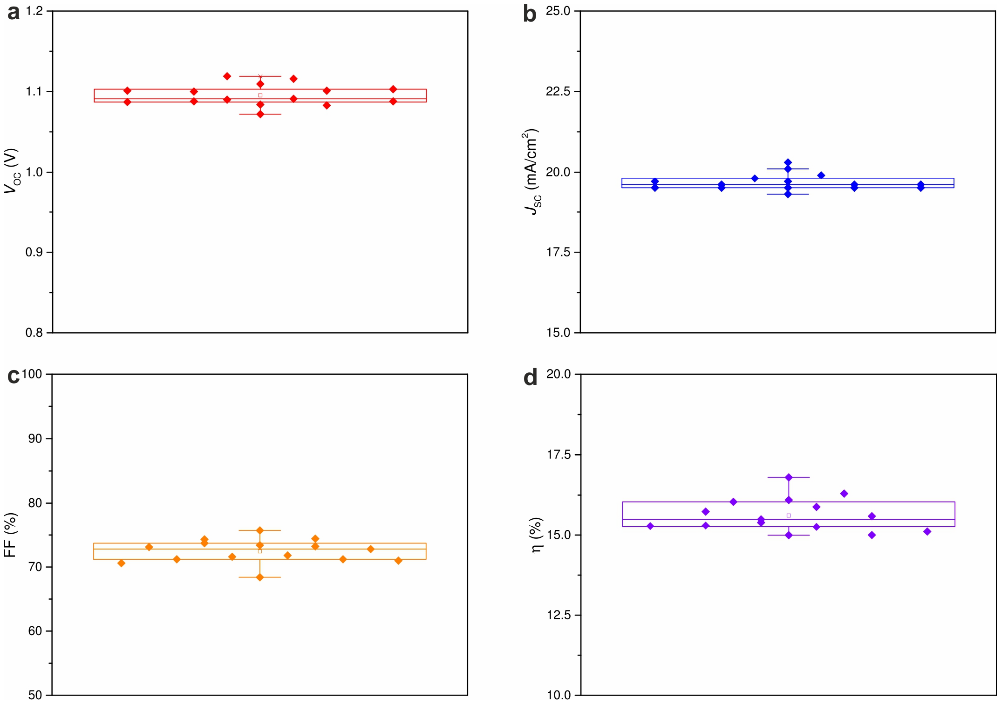

Figure S5. Statistics of device performance with graded absorber. a-d, The V_{OC} (**a**), J_{SC} (**b**), FF (**c**), and η (**d**) distribution of 15 graded perovskite solar cells, which yields an average efficiency of 15.6%.

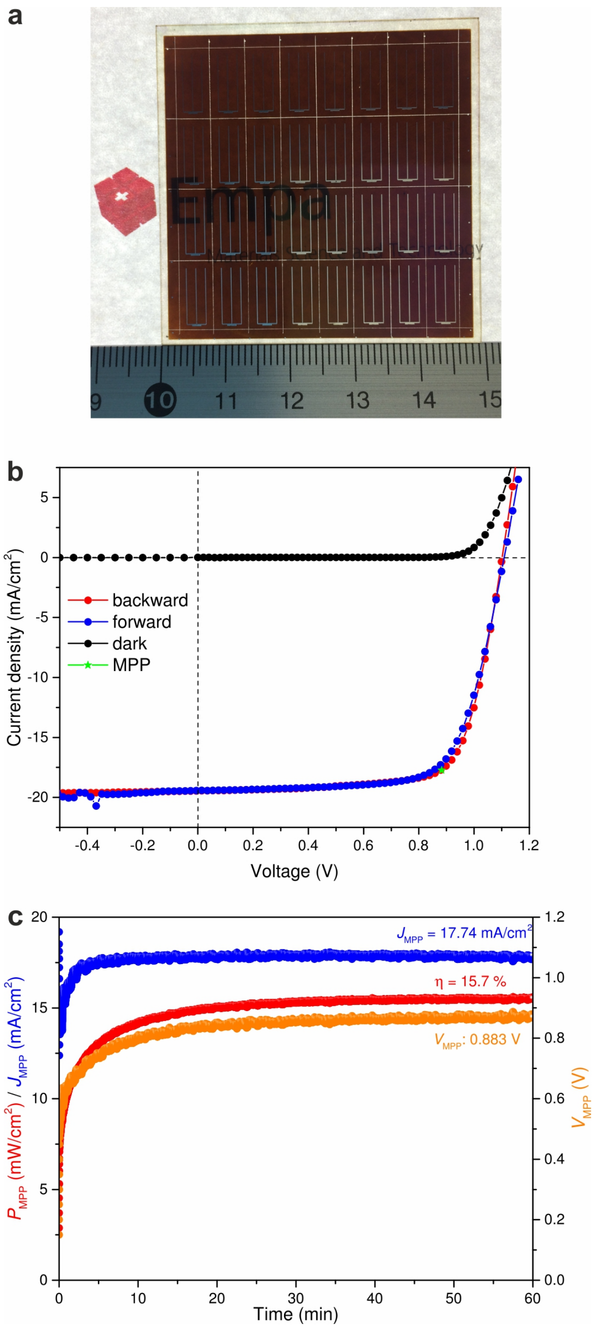

Figure S6. Photovoltaic performance of NIR-transparent $MAPbI_{3-x}Br_x$ perovskite solar cells grown on 5 cm × 5 cm glass substrate. a, The photograph of graded perovskite solar cells grown on a 5 cm × 5 cm substrate. **b, c,** The *J-V* curves (**b**) and MPP measurement (**c**) of a 15.7% efficient NIR-transparent graded perovskite solar cell with a cell size of 0.561 cm^2.

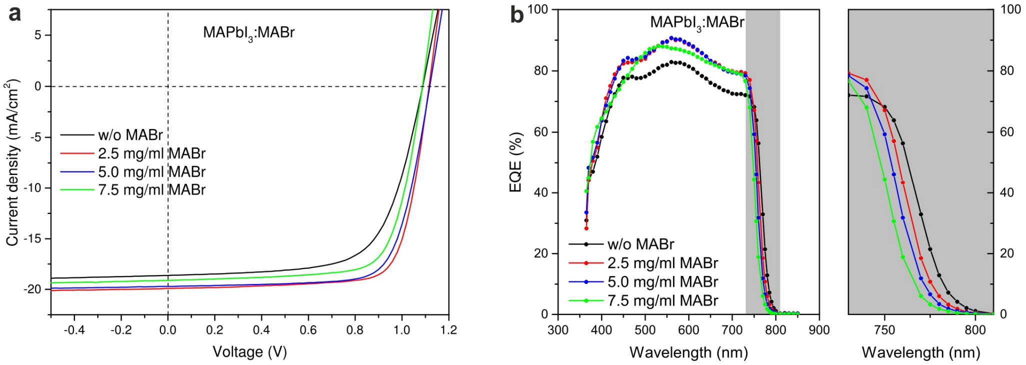

Figure S7. Photovoltaic performance of graded perovskite solar cells prepared with various MABr concentration. a, b, The *J-V* curves (**a**) and external quantum efficiency (EQE) spectra (**b**) of graded $MAPbI_{3-x}Br_x$ perovskite solar cells prepared with various MABr concentrations.

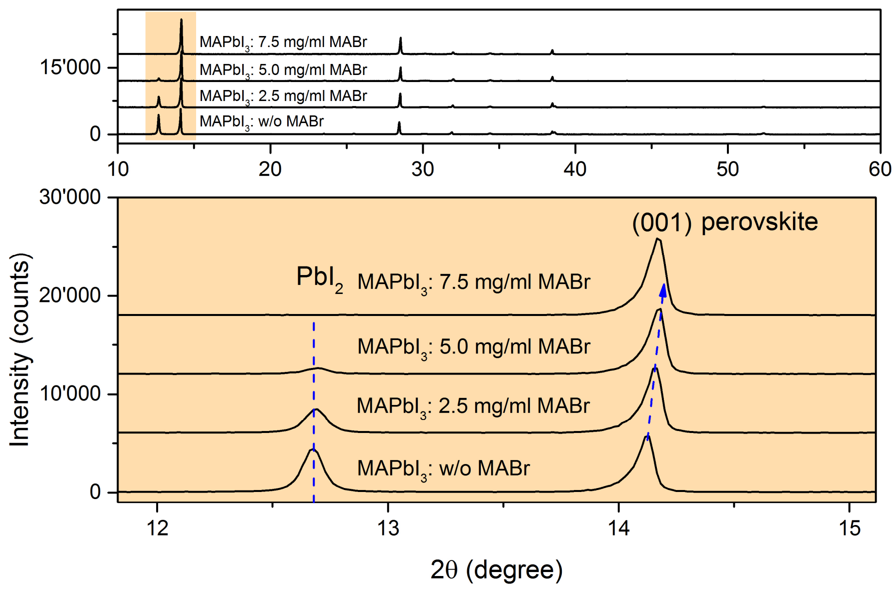

Figure S8. X-ray diffraction (XRD) patterns of NIR-transparent devices with $MAPbI_3$:MABr absorbers. The perovskite (001) peak shifted towards higher angle with increasing MABr concentration, indicating I atoms were replaced by smaller Br in the lattice.

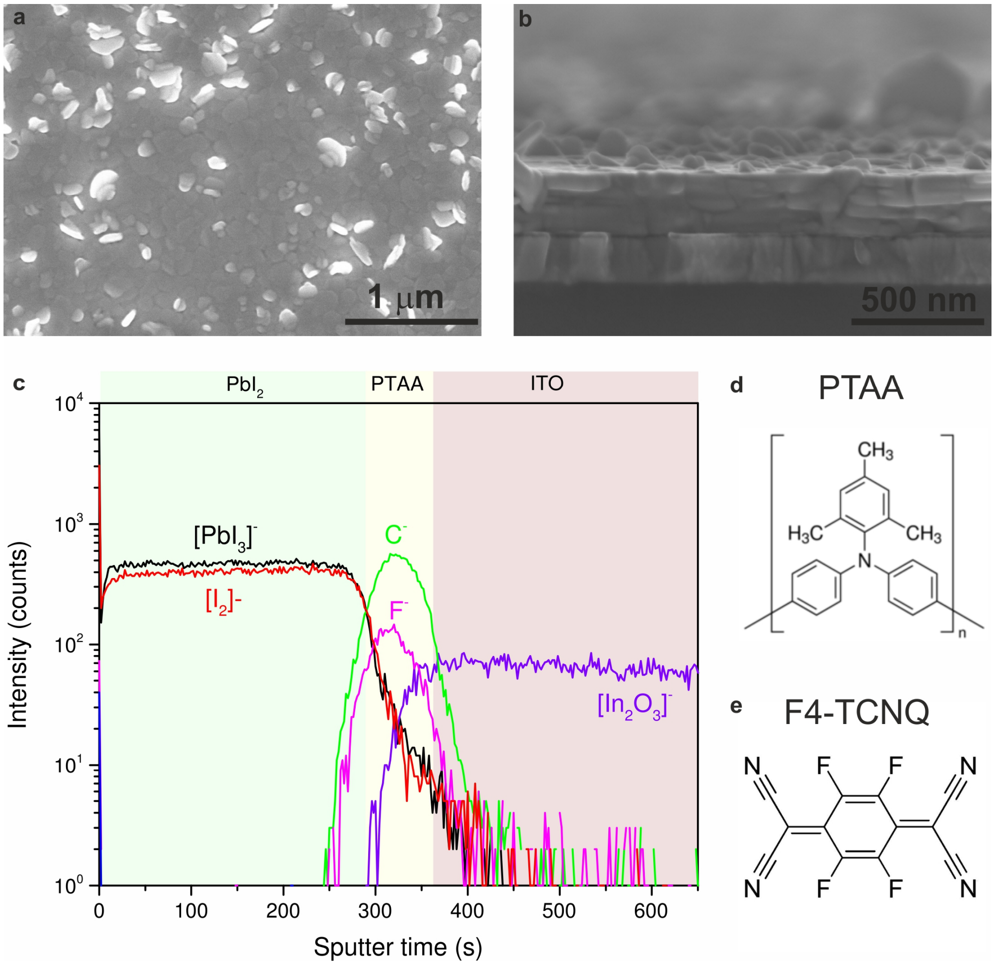

Figure S9. Thermally evaporated PbI_2 layer on PTAA. a, b, c, The top view (**a**) and cross-sectional (**b**) SEM images, and the corresponding SIMS depth profile (**c**) of PbI_2 film grown on PTAA/ITO/glass substrate. **d, e,** The structure of PTAA (**d**) and F4-TCNQ (**e**). The PbI_2 prepared by thermal evaporation exhibits compact morphology. In the ToF-SIMS depth profile, $[PbI_3]^-$, and $[I_2]^-$ ions are used to represent the PbI_2 layer, and C^-, F^- are used to label the PTAA (doped with 1wt% F4-TCNQ) layer, and $[In_2O_3]^-$ is chosen to refer to the ITO layer.

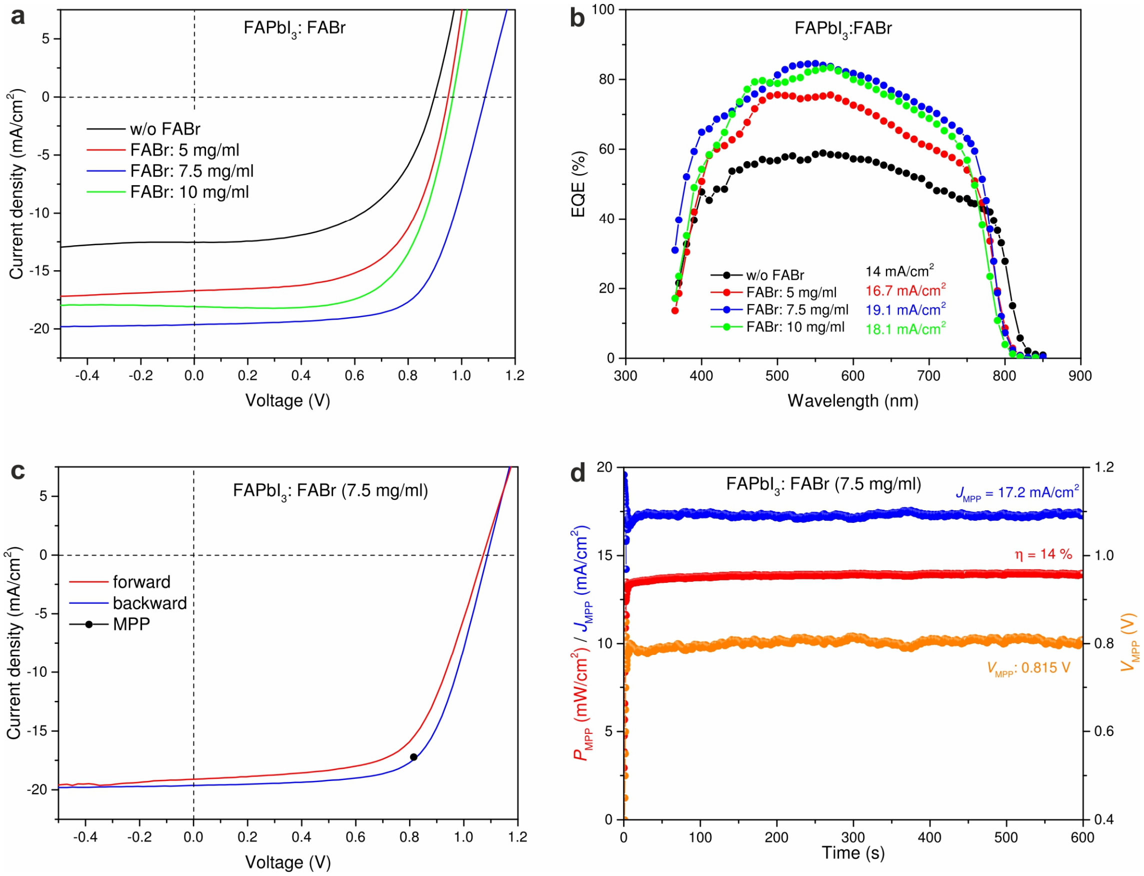

Figure S10. Photovoltaic performance of graded $FAPbI_3$:FABr perovskite solar cells. a, b, The *J*-*V* curves (**a**) and external quantum efficiency (EQE) spectra (**b**) of graded $FAPbI_3$: MABr perovskite solar cells prepared with various FABr concentraiton. **c, d,** The *J*-*V* curves (**c**) and MPP measurement (**d**) of the best performing graded $FAPbI_3$:FABr (7.5 mg ml^{-1}) devices. Although a slight hysteresis is observed for $FAPbI_3$ based devices, the MPP lies on the backward scan.

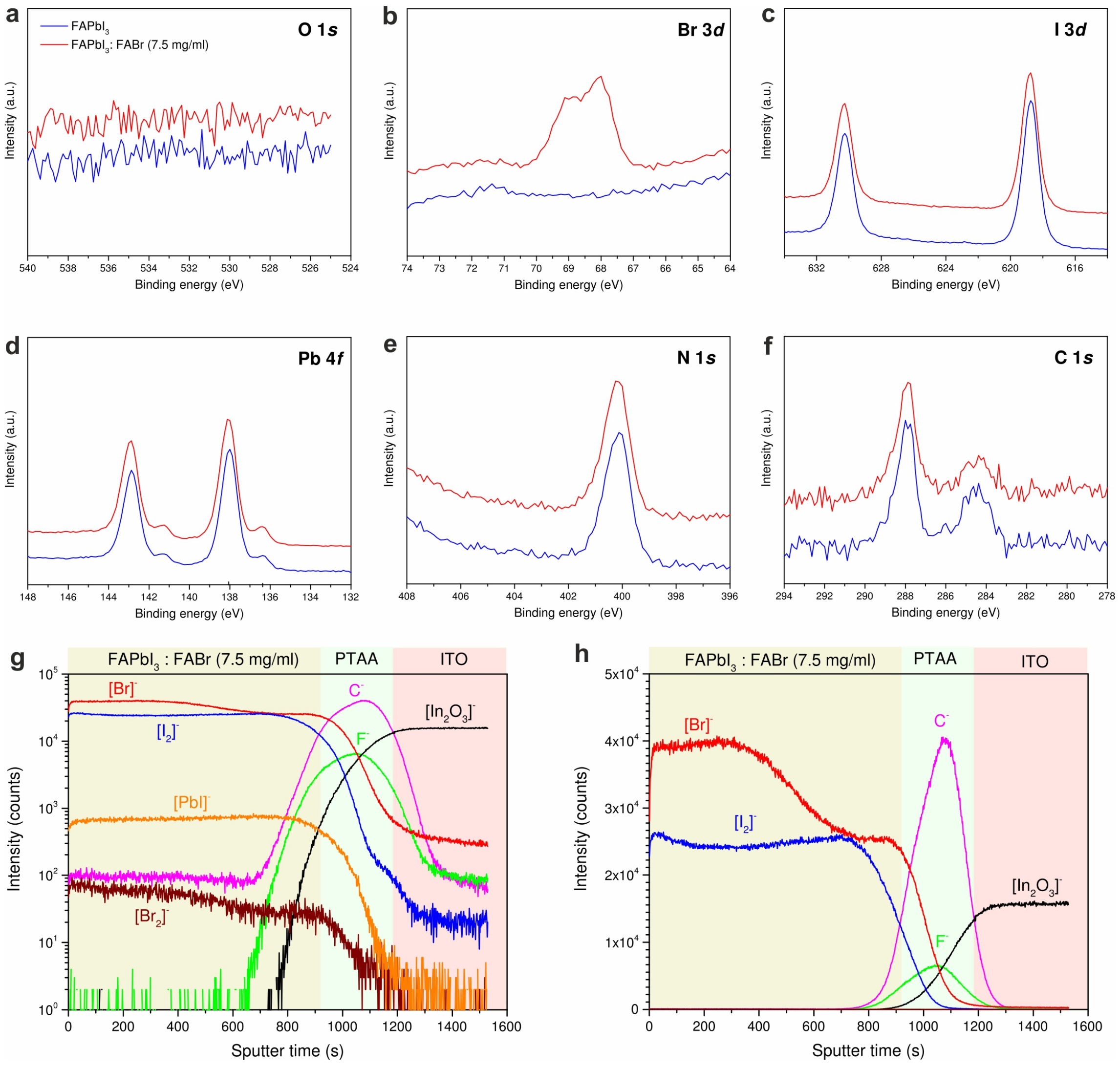

Figure S11. Surface composition and depth profile of graded $FAPbI_3$:FABr (7.5 mg/ml) absorber. a – f, The X-ray photoelectron spectroscopy (XPS) O 1*s* (**a**), Br 3*d* (**b**), I 3*d* (**c**), Pb 4*f* (**d**), C 1*s* (**e**), N 1*s* (**f**) core-level spectra for reference ($FAPbI_3$) and graded absorber ($FAPbI_3$: FABr (7.5 mg/ml)). **g – h,** The ToF-SIMS depth profile of graded $FAPbI_3$: FABr (7.5 mg/ml) absorber shown in logarithm (**g**) and linear (**h**) scale, respectively. The Br gradient is clearly detected in the depth profile.

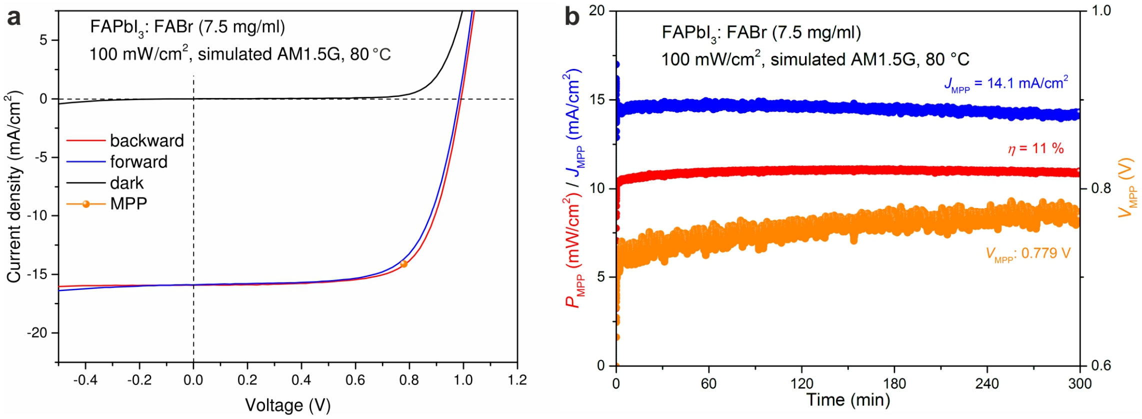

Figure S12. High temperature photovoltaic performance of perovskite solar cells with graded $FAPbI_3$:FABr (7.5 mg/ml) absorber. a, b, The *J*-*V* curves (**a**) and maximum power point (MPP) measurement (**b**) were conducted at 80 °C in ambient air with 50% relative humidity. The *J*-*V* and MPP measurement were performed under simulated AM1.5G one-sun illumination. The device efficiency is stable during the 5 hours MPP operation at 80 °C in ambient air, indicating promising thermal- and photo-stability.

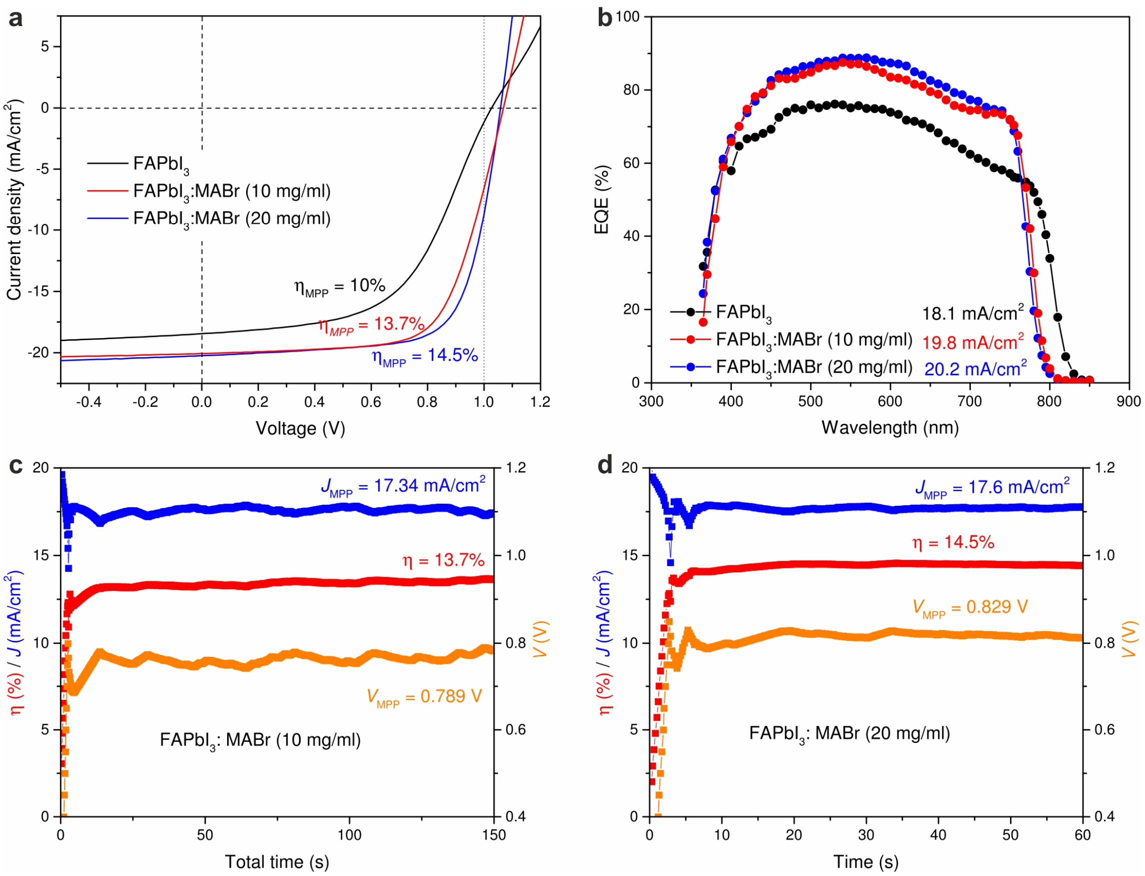

Figure S13. Photovoltaic performance of graded $FAPbI_3$:FABr perovskite solar cells. a, b, The *J-V* curves (**a**) and external quantum efficiency (EQE) spectra (**b**) of graded $FAPbI_3$:MAB perovskite solar cells prepared with different MABr concentration. **c, d,** The MPP measurement for $FAPbI_3$:MABr (10 mg ml^{-1}) (**c**) and $FAPbI_3$:MABr (20 mg ml^{-1}) (**d**) devices. The bandgap shifted over 0.1 eV after ion-exchange with MABr.

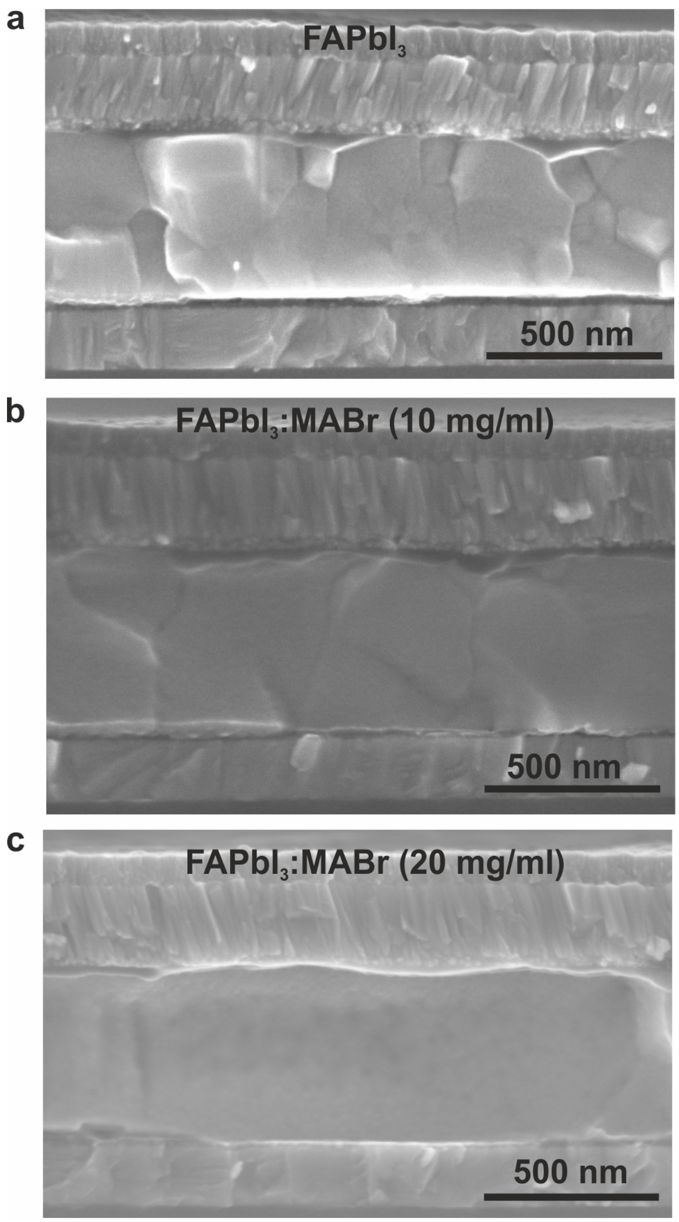

Figure S14. Microstructure of $FAPbI_3$ based graded perovskite solar cells. a, b, c, The cross-sectional SEM images of NIR-transparent perovskite solar cells with reference $FAPbI_3$ absorber (**a**), and graded $FAPbI_3$: MABr (10 mg/ml) (**b**) and $FAPbI_3$: MABr (20 mg/ml) (**c**) absorbers. The absorber thickness in all devices is around 520 nm. The device structure is glass/ITO/PTAA/perovskite/PCBM/ZnO nanoparticles/AZO/Ni-Al grid/MgF_2.

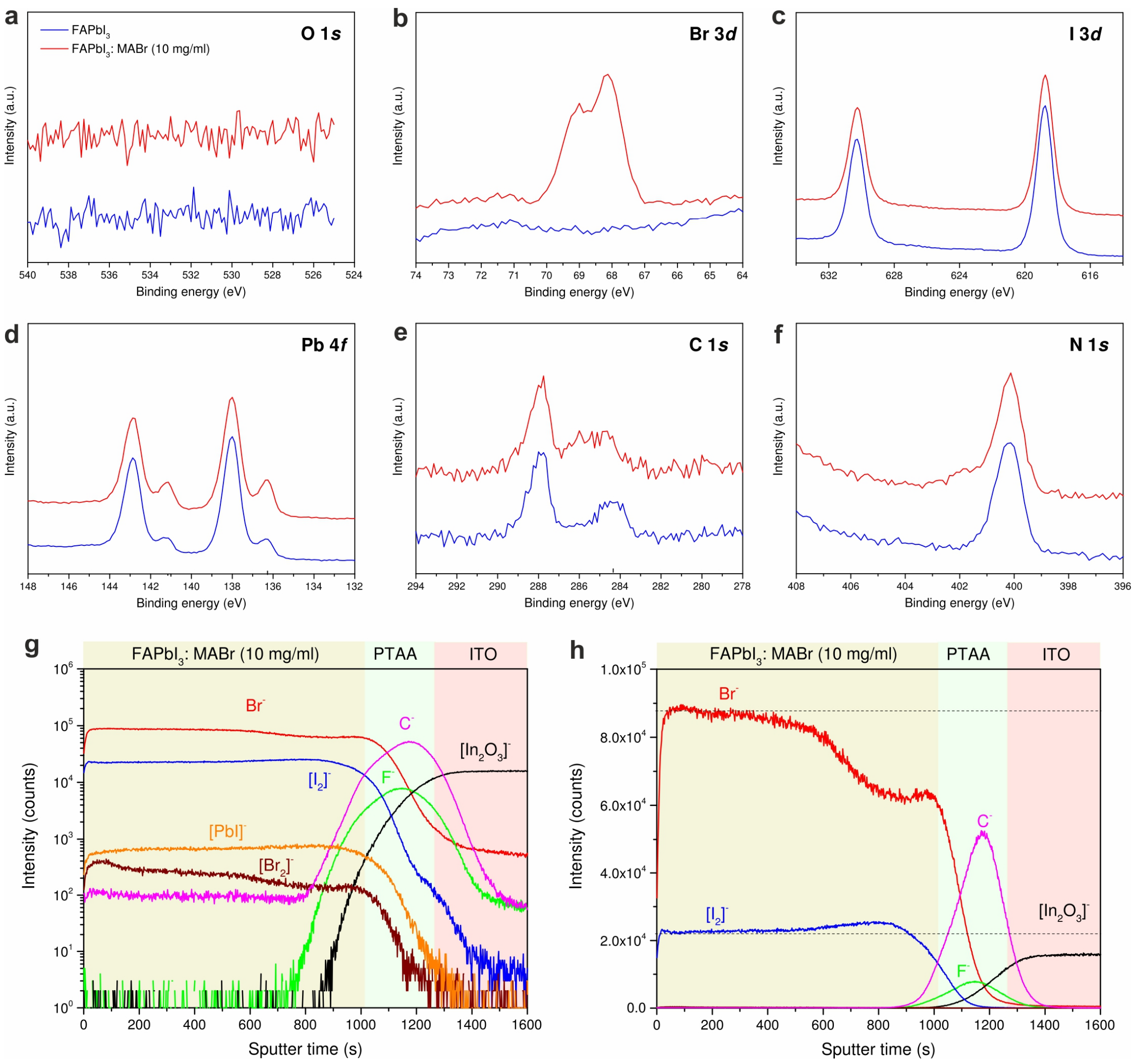

Figure S15. Surface composition and depth profile of graded $FAPbI_3$:MABr (10 mg/ml) absorber. a – f, The X-ray photoelectron spectroscopy (XPS) O 1*s* (**a**), Br 3*d* (**b**), I 3*d* (**c**), Pb 4*f* (**d**), C 1*s* (**e**), N 1*s* (**f**) core-level spectra for reference (bare $FAPbI_3$) and graded absorber ($FAPbI_3$: MABr (10 mg/ml)). **g – h,** the ToF-SIMS depth profile of graded $FAPbI_3$: MABr (10 mg/ml) absorber shown in logarithm (**g**) and linear (**h**) scale. The Br gradient is clearly detected in the depth profile.

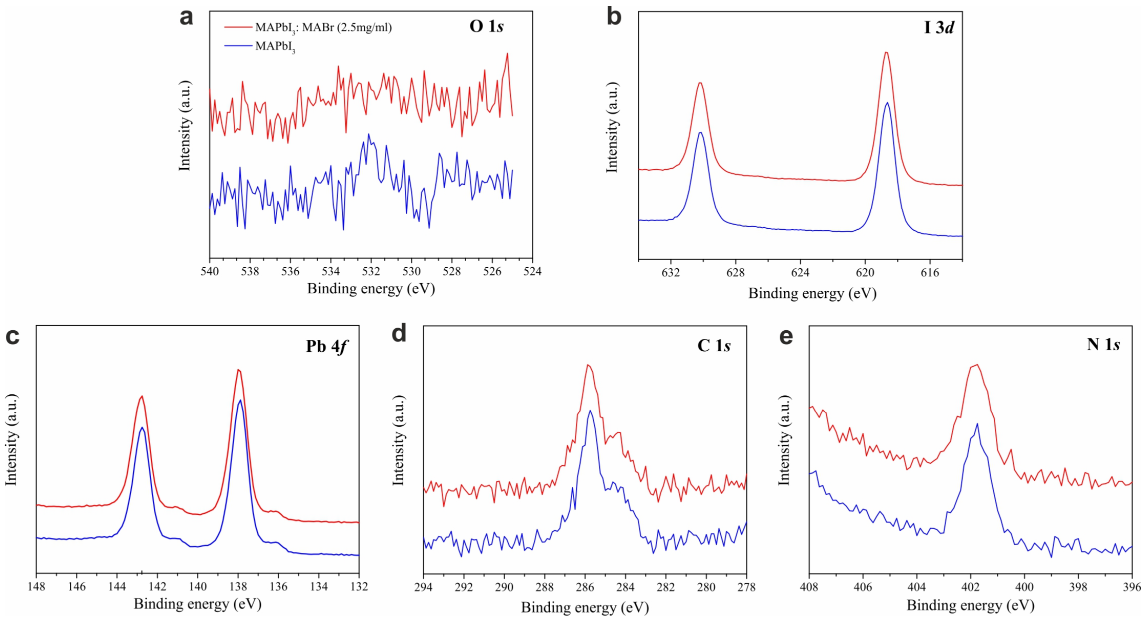

Figure S16. Surface composition of graded $MAPbI_3$:MABr (2.5 mg ml^{-1}) absrober. a-e, X-ray photoelectron spectroscopy (XPS) for O 1*s* (**a**), I 3*d* (**b**), Pb 4*f* (**c**), C 1*s* (**d**), N 1*s* (**e**) core-level spectra for graded absorber ($MAPbI_3$:MABr 2.5 mg ml^{-1}) and reference (bare $MAPbI_3$). The O 1*s* peak at around 532 eV is negligible in both samples, indicating minimum contamination during the transfer of samples from the glovebox to the XPS chamber.

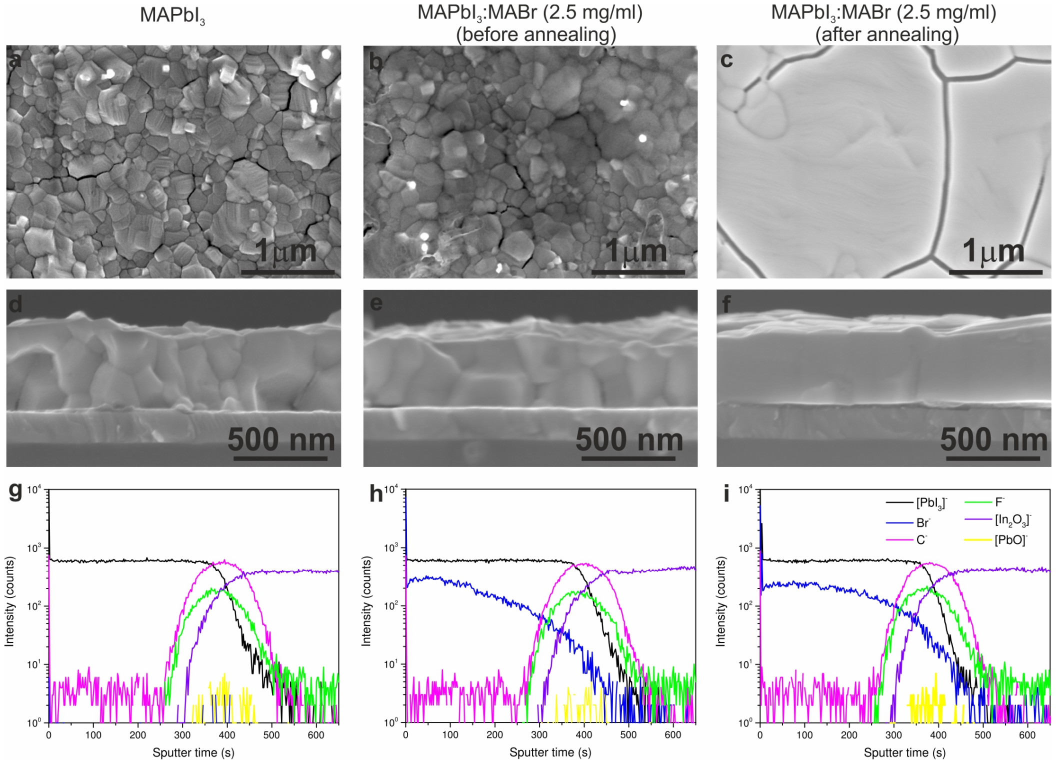

Figure S17. Morphology and composition evolution during PIE. a-f, The SEM images of the starting absorber (**a**, **d**), as-spun intermediate absorber (**b**, **e**), and final absorber (**c**, **f**). The top view and cross-sectional SEM images (**a**, **b**, **d**, **e**) did not show appreciable morphology and microstructure changes after spin coating, while the grain size increased dramatically after chlorobenzene vapor assisted thermal annealing (**c**, **f**). **g-i**, The time-of-flight secondary ion mass spectrometry (ToF-SIMS) elemental depth profiling of the corresponding starting absorber (**g**), intermediate absorber (**h**), and final absorber (**i**). $[PbI_3]^-$, Br^- are used to represent perovskite layer, C^- and F^- are chosen to indicate PTAA (doped with F4-TCNQ) layer, and $[In_2O_3]^-$ is employed to label ITO layer. The absence of $[PbO]^-$ signal in the perovskite layer indicates that samples are not contaminated during sample preparation and transportation. In the starting absorber (**g**), the perovskite/PTAA/ITO layer structure can be clearly observed. After ion-exchange with the MABr solution (**h**), the Br signal was already detected at the front interface and a strong gradient towards rear interface was observed. This suggested the ion-exchange mainly occurred at the front interface. After chlorobenzene vapor annealing, the Br intensity decreased at the front interface and increased at the rear interface, implying an ion diffusion and redistribution after annealing (**i**). Most importantly, the Br gradient remained in the final absorber.

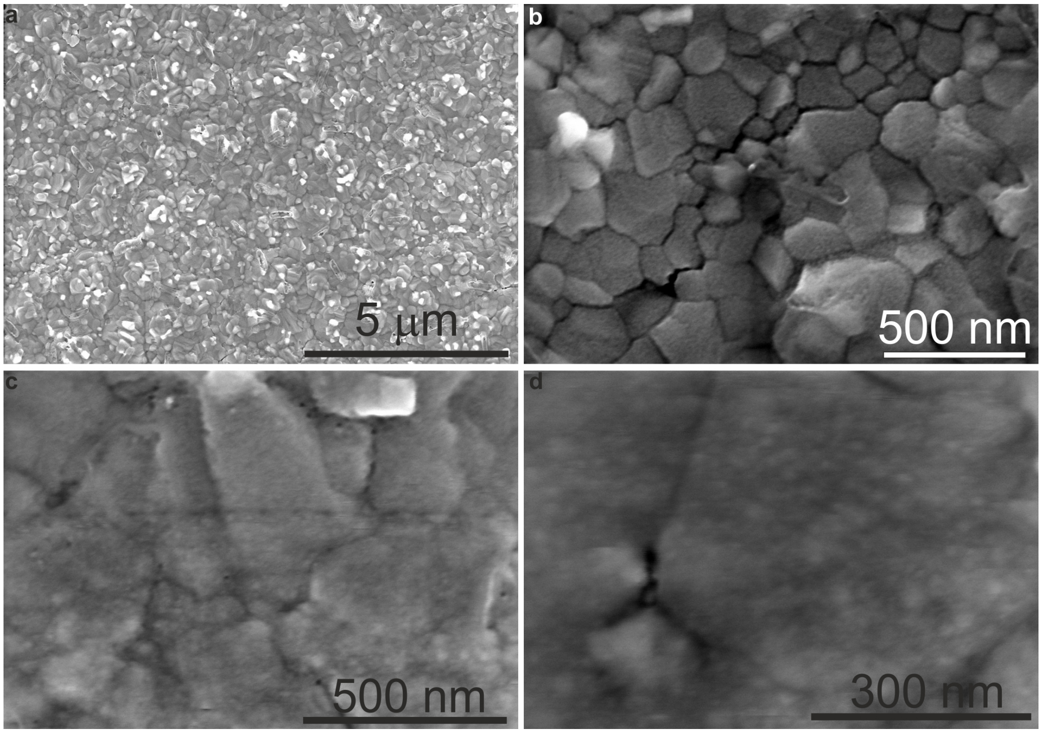

Figure S18. Morphology of perovskite layer after halide ion-exchange. a-d, The morphology of a perovskite film after spin coating of MABr (2.5 mg ml^{-1}) on a starting $MAPbI_3$ absorber. The low magnification SEM image (**a**) does not show an obvious difference from starting absorber; however, the high magnification images (**b-d**) reveal nanostructured morphology, suggesting strong chemical reaction occurred on the surface of the absorber. These results support the hypothesis that ion-exchange mainly occurred during spin coating.

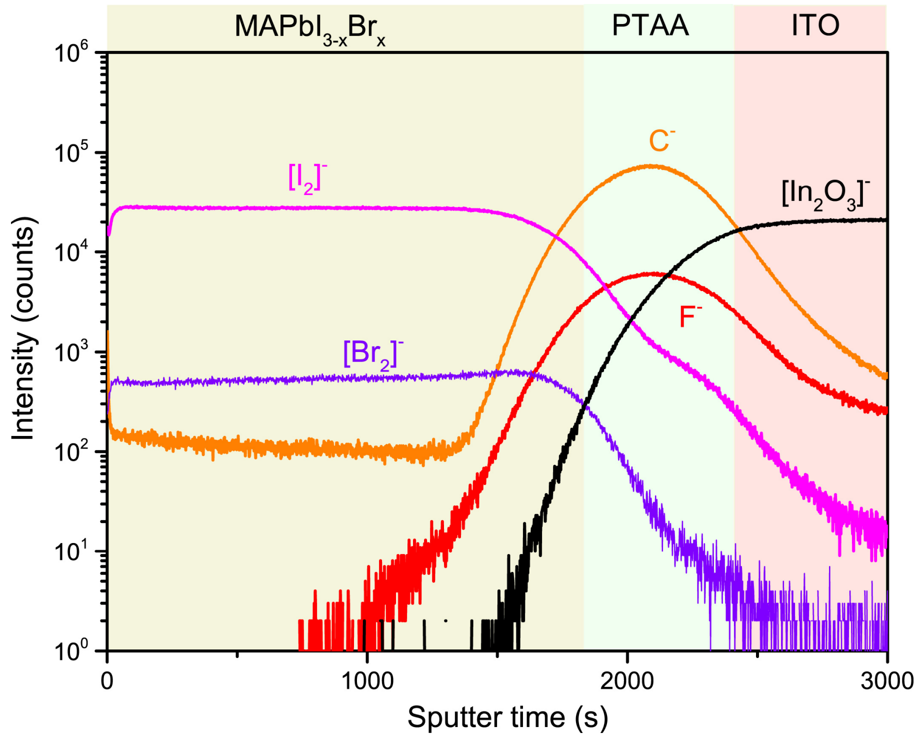

Figure S19. ToF-SIMS depth profile of mixed-halide perovskite prepared by spin coating blended MAI/MABr solution onto PbI_2. The Br gradient is not observed when a mixed MAI/MABr solution is employed.

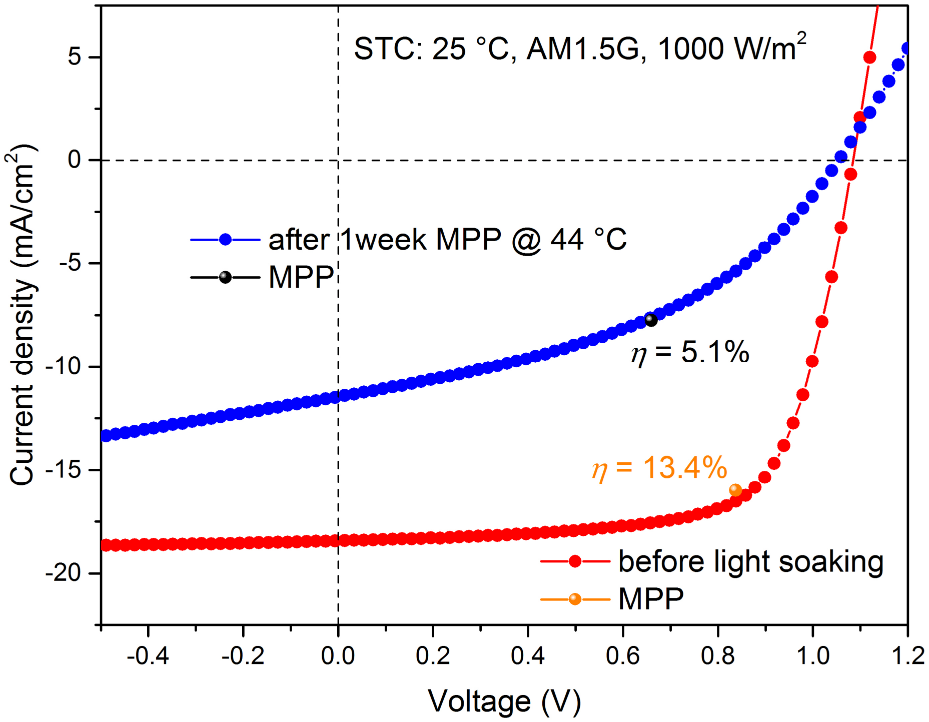

Figure S20. Device stability for reference $MAPbI_3$ solar cell. The device was stressed together with a Br-graded cell under same condition. After continuous operation at MPP condition for 1 week at 44 °C, the device was taken out of the stress chamber and characterized under STC. The reference cell already lost over 60% of its initial performance after 1 week MPP operation at 44 °C. Due to the low performance, the cell was not further stressed at 60 and 80 °C.

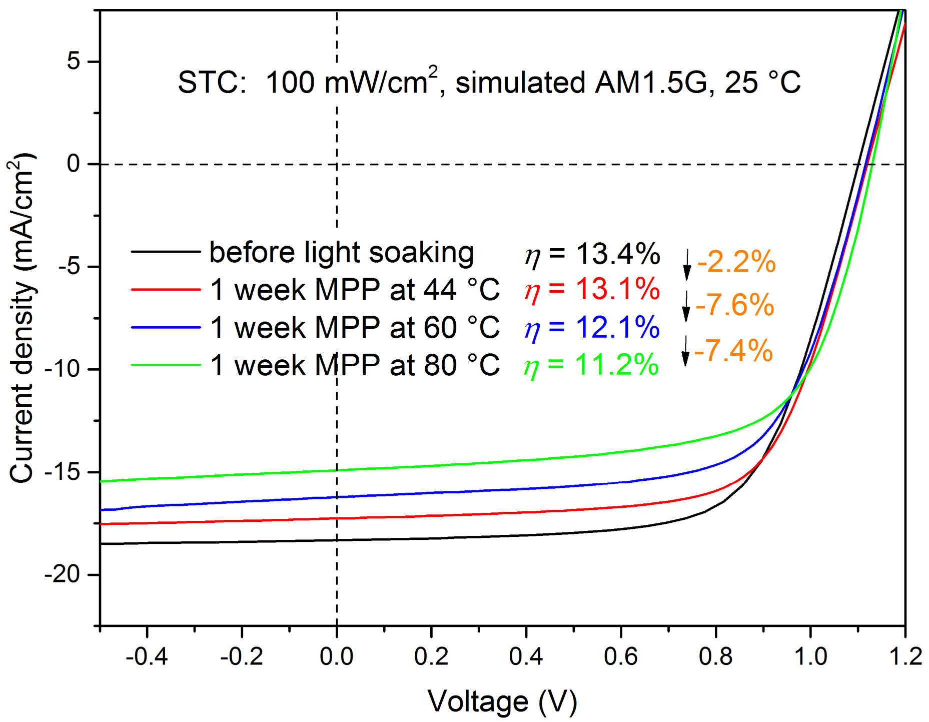

Figure S21. Device stability for graded $MAPbI_3$:MABr (2.5 mg ml^{-1}) solar cell. A non-encapsulated graded device was stressed at MPP condition under continuous equivalent one-sun illumination for 1 week at 44, 60, and 80 °C, respectively. The device was taken out of the stress chamber after one week stress at each temperature to conduct *J-V* characterization under STC. The graded device retained over 92% of its initial efficiency after each stress cycle, indicating significantly improved operational stability compared to the reference $MAPbI_3$ device without Br.

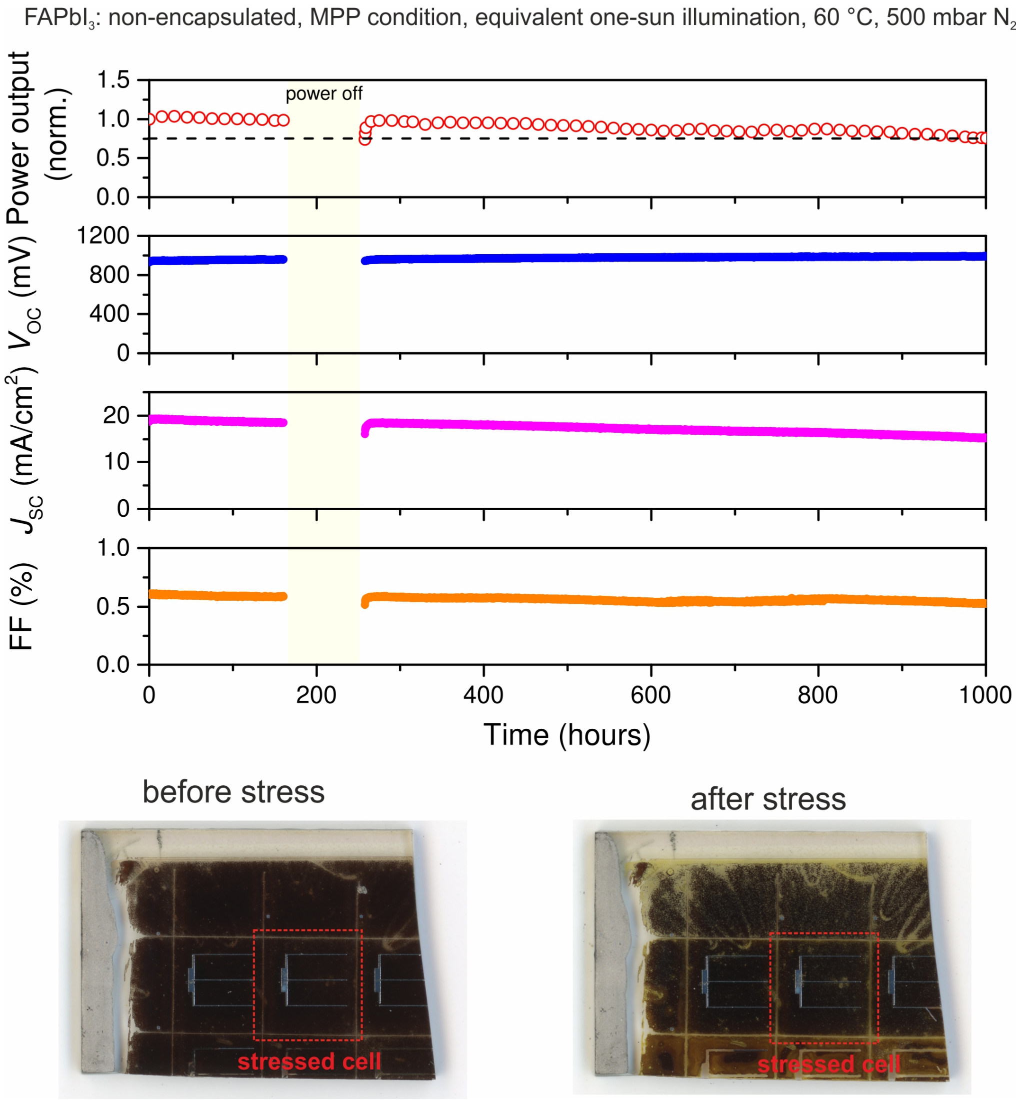

Figure S22. The photovoltaic parameters of a reference $FAPbI_3$ cell during heat/light soaking test. The stress condition was the same as for the graded $FAPbI_3$:MABr (10 mg ml^{-1}) cell. The reference cell only retained 75% of its initial efficiency after the heat/light soaking test, mainly due the decrease in J_{SC} and FF. As can be seen from the photograph of the cell after stress test, the J_{SC} loss probably mainly comes from the decomposition of perovskite.

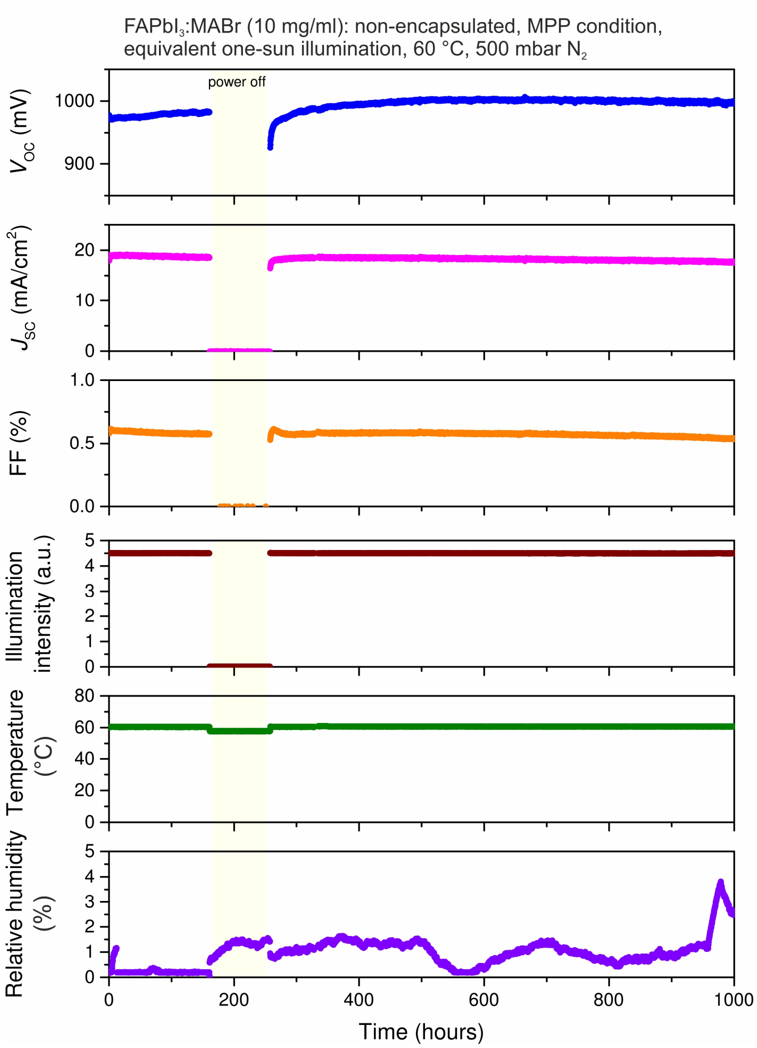

Figure S23. The photovoltaic parameters of graded $FAPbI_3$:MABr (10 mg ml^{-1}) during heat/light soaking test. A non-encapsulated device was kept at MPP condition for 1000 hours under equivalent one-sun illumination at 60 °C. White light emitting diodes (LED) are used as a light source, which does not contain UV and IR light. The device was exposed to full area illumination under 500 mbar N_2 atmosphere. A *J*-*V* curve was recorded *in-situ* every 15 min. The graded cell was already light soaked under 1-sun illumination for over 120 hours at 44 °C before the stability test. We note that the illumination was unintentionally off during the stability test due to the errors in power supply. After turning on the light again, V_{OC}, J_{SC} and FF restored to values before light off with a slow transient. As the temperature was still kept at around 60°C, the light soaking effect was induced by light illumination rather than temperature. During the 1000 hours heat/light soaking test, the V_{OC} gradually increased to over 1 V, while J_{SC} and FF decreased slightly, resulting in less than 7% loss in efficiency.

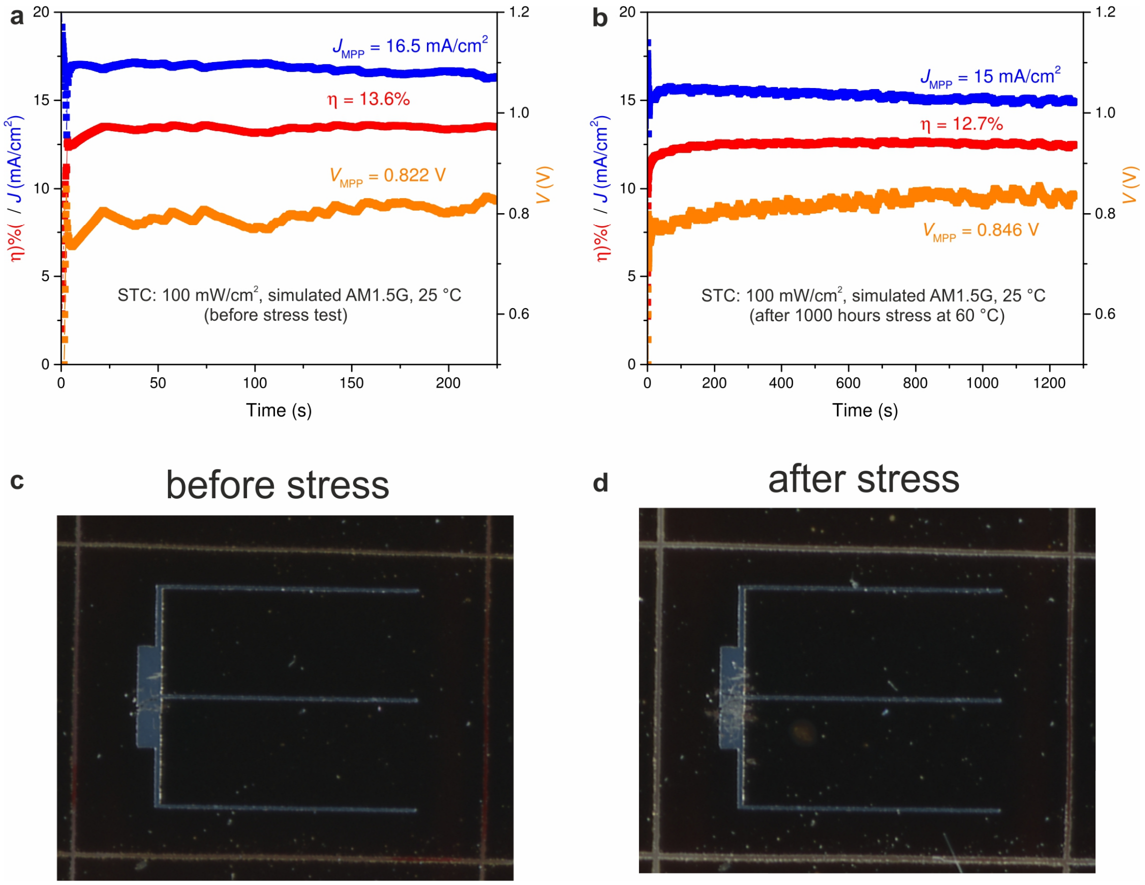

Figure S24. Steady state efficiency of graded $FAPbI_3$:MABr (10 mg ml^{-1}) device measured under STC. a, **b**, The MPP measurement of a graded device measured before (**a**) and after (**b**) heat/light soaking under STC. **c, d,** The comparison of photographs of stressed devices before (**c**) and after (**d**) the heat/light soaking test. After 1000 hours stress at 60 °C, yellow spots appeared near the grid finger and scribed line of the cell, which decreased the active area.

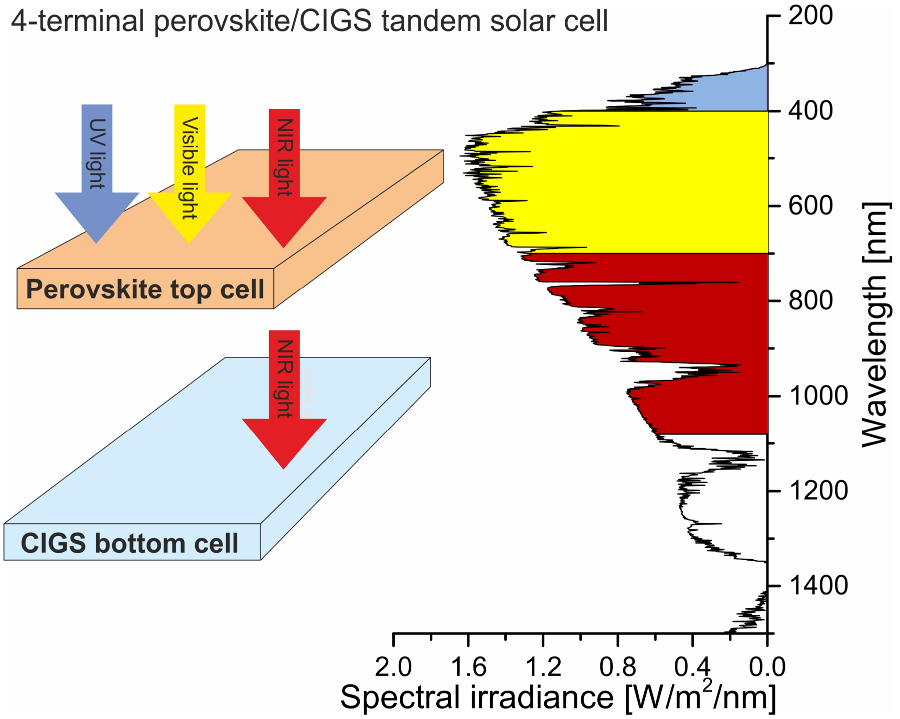

Figure S25. Schematic of 4-terminal Perovskite/CIGS thin film tandem solar cells. The perovskite top cell and CIGS bottom cell are optically coupled and electrically de-coupled. Therefore, the top cell and bottom cell can be individually processed and then mechanically stacked on top of each other. In this configuration, the perovskite top cell and CIGS bottom cell can both operate at their respective MPP and the addition of these efficiencies gives the 4-terminal tandem efficiency. Compared to single absorber solar cell, the thermalization losses are significantly reduced in tandem solar cells.
